# Joint effects of childhood adversity and genetic risk for psychosis on psychopathology in the UK Biobank

**DOI:** 10.1017/S0033291726104012

**Published:** 2026-04-07

**Authors:** Zheng-An Lu, Alexander Ploner, Sarah E. Bergen

**Affiliations:** Medical Epidemiology and Biostatistics, Karolinska Institute, Stockholm, Sweden

**Keywords:** schizophrenia, Bipolar Disorder, psychopathy, genetic risk, Adverse Childhood Experiences

## Abstract

**Background:**

The individual effects of genetic factors and adverse childhood experiences (ACEs) on risk of psychosis, including schizophrenia (SCZ) and bipolar disorder (BIP), have been widely acknowledged, but their interaction effects on individual psychopathological symptoms remain unclear.

**Methods:**

Based on data from 163,704 individuals in the UK Biobank, we investigated the joint effects of polygenic risk scores (PRSs) of SCZ and BIP and ACEs on psychopathology. ACEs status and 55 psychopathological symptoms from seven domains were measured retrospectively using an online mental health questionnaire in 2016. Recent genome-wide association studies for SCZ and BIP were combined with genotype data to generate PRSs. Logistic regression analyses were then conducted to explore univariate and joint main effects of PRSs and ACEs on psychopathological symptoms, as well as their additive and multiplicative interaction effects.

**Results:**

The interaction mechanisms for PRSs and ACEs varied across symptom domains: additive interactions were observed on the depression (RERI_BIP-ACEs_ = 0.20–0.25), anxiety (RERI_SCZ-ACEs_ = 0.20; RERI_BIP-ACEs_ = 0.22–0.26), help-seeking (RERI_SCZ-ACEs_ = 0.24; RERI_BIP-ACEs_ = 0.23), and cognition domains (RERI_SCZ-ACEs_ = −0.23 to -0.17), whereas multiplicative interactions were only detected on the psychotic (beta_SCZ-ACEs_ = −0.543; beta_BIP-ACEs_ = −0.181), mania (beta_BIP-ACEs_ = −0.195), self-harm or suicide (beta_SCZ-ACEs_ = −0.118), and cognitive domains (beta_SCZ-ACEs_ = −0.204 to −0.157).

**Conclusions:**

The interplay mechanisms for genetic liability to SCZ and BIP and ACEs vary across symptom domains. This study reveals heterogeneity in gene–ACEs interaction mechanisms underlying psychosis and may provide personalized guidance for psychological care after ACEs.

## Introduction

Psychosis, including schizophrenia (SCZ) and bipolar disorder (BIP), is severe and heterogeneous with substantial disease burden and few effective treatments, affecting ∼0.7 and ∼1.0% of the world’s population, respectively (McGrath, Saha, Chant, & Welham, 2008; Merikangas et al., 2011). SCZ and BIP demonstrate considerable overlap in terms of symptomatology, etiology, outcomes, and treatment responses (Yamada, Matsumoto, Iijima, & Sumiyoshi, 2020).

It is widely acknowledged that genetic foundations play an important role in the onset of psychosis, with twin-based heritability estimated at 60–80% for SCZ and 80–90% for BIP (Hilker et al., 2018; O’Connell & Coombes, 2021). Genetic risk for these disorders indexed by polygenic risk scores (PRSs) explains ∼9% of the variance in SCZ and ∼22% of the variance in BIP, respectively (Calafato et al., 2018; O’Connell et al., 2025). Moreover, some of the contributions of these PRSs to psychopathology are non-specific and transdiagnostic, as they explain 1–3% of the variance for other mental disorders (Calafato et al., 2018). PRSs have limited predictive power and only index risk from common genetic variants, but their strong associations with SCZ or BIP diagnoses support their utility as a research tool (Smeland & Andreassen, 2021).

Adverse childhood experiences (ACEs) are also major drivers for psychosis (Robinson & Bergen, 2021; Varese et al., 2012). ACEs are defined as traumatic events happening in childhood with potentially long-term negative impacts, which include physical/emotional/sexual abuse, neglect, discrimination, parental death, or separation (Köhler-Forsberg et al., 2024). ACEs are found to be associated with risk of SCZ and BIP in a dose–response manner: exposure to a higher number of ACEs is associated with a greater risk of SCZ or BIP, as well as more severe symptoms, worse psychological functioning, and more adverse long-term outcomes among individuals with SCZ and BIP (Köhler-Forsberg et al., 2024; Peralta et al., 2024; Yao, van der Veen, Thygesen, Bass, & McQuillin, 2023; Zhang et al., 2023).

Despite the widely acknowledged individual effects of genetic factors and ACEs, their joint contribution remains largely unclear (Woolway et al., 2022). Previous studies have produced inconsistent findings: some suggest that PRSs and ACEs are independently associated with psychosis risk in joint models, but find no evidence for their interactions (Tonini et al., 2022; Trotta et al., 2016). Other studies report additive synergistic effects, where the combined effect of polygenic risk and ACEs on psychosis was greater than the sum of each alone (Aas et al., 2023). Furthermore, a multiplicative interaction effect has been observed, which was reflected by a differential effect of ACEs on psychopathology stratified by individuals with various genetic liabilities, although this was not replicated in other studies (Alameda et al., 2024).

One potential explanation for these mixed findings is the considerable heterogeneity in symptomatology of SCZ and BIP (Dacquino, De Rossi, & Spalletta, 2015). Despite specific diagnostic criteria, all common psychopathological domains, including psychotic experiences, mania, depression, anxiety, self-harm or suicide, and cognitive impairment, can manifest in both SCZ and BIP (Dacquino et al., 2015). Help-seeking is also an important prognostic factor for both SCZ and BIP, as a delay in seeking help leads to adverse clinical outcomes and negative responses to care (Clement et al., 2015). Moreover, both polygenic risk and ACEs contribute to the general psychopathology and help-seeking behaviors in a transdiagnostic manner (Calafato et al., 2018; Daníelsdóttir et al., 2024; Karatekin, 2019; Rayner et al., 2019). Most existing studies examining the joint contributions of polygenic risk and ACEs focus on their impacts on clinical diagnoses or overall symptom severity. However, if the size and direction of the respective contributions from PRSs and ACEs, as well as their interaction mechanisms, vary across individual symptoms, mixed findings can emerge at these global scales. Investigating the mechanisms by which polygenic risk for SCZ and BIP and ACEs jointly contribute to individual psychopathological symptoms can therefore deepen our understanding of the underlying etiology of psychosis.

This study aimed to comprehensively examine the joint contribution of polygenic risk for SCZ or BIP and ACEs to psychopathological symptoms across seven symptom domains based on approximately 160,000 individuals from the UK Biobank. We hypothesized that the interaction mechanisms for polygenic risk and ACEs might vary across different psychopathological symptom domains. Specifically, we tried to answer the following four research questions: (1) which psychopathological symptoms are associated with PRS for SCZ, PRS for BIP, and ACEs individually; (2) whether psychopathological symptoms can be independently predicted by PRSs for SCZ or BIP and ACEs in a joint main effect model; (3 + 4) whether PRSs for SCZ or BIP and ACEs exert additive (3) or multiplicative (4) interactions on these symptoms.

## Methods

### Participants

**Figure 1.**
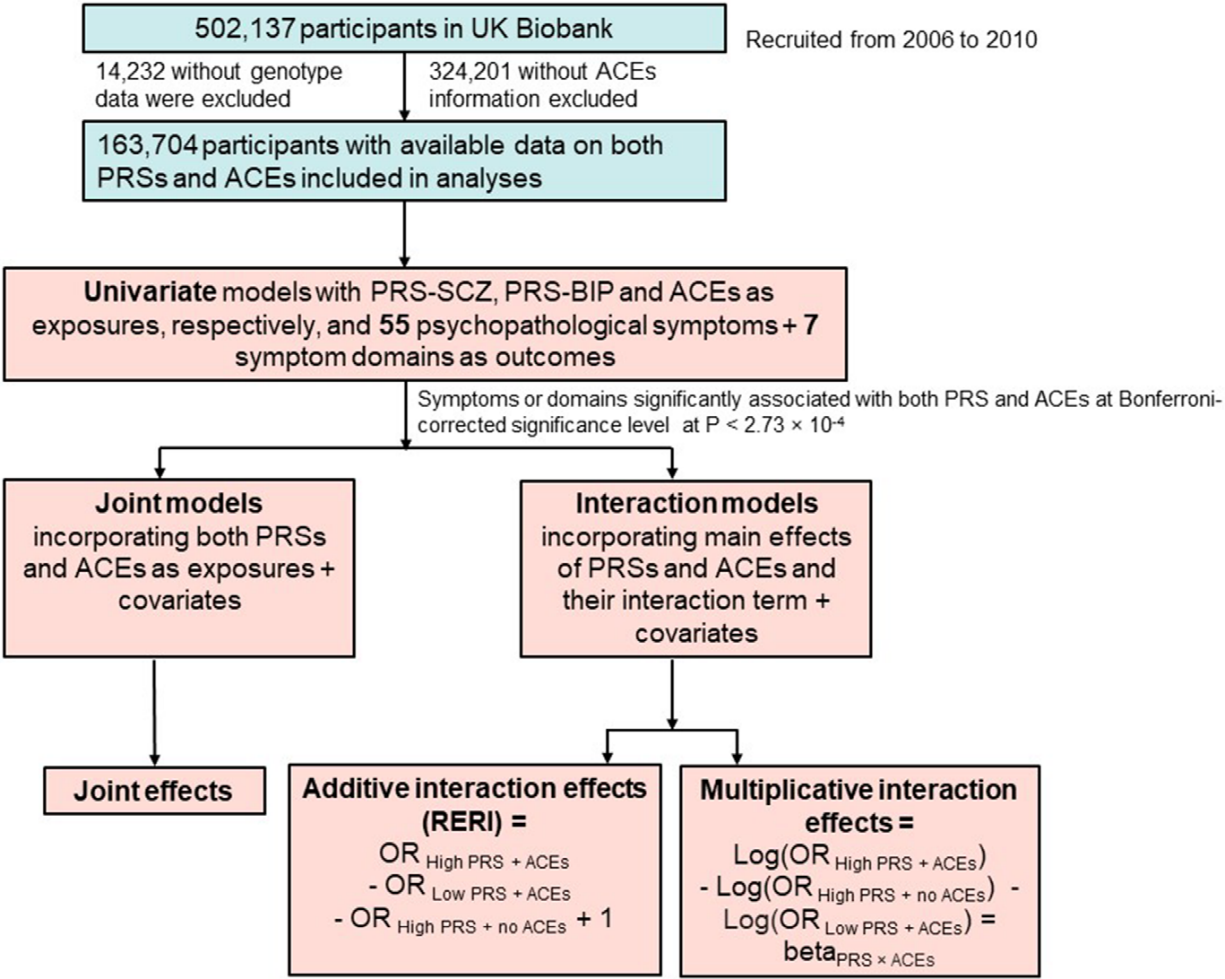
Flow chart of the current study. ACEs, adverse childhood experiences; OR, odds ratio; PRSs, polygenic risk scores.

All participants in our study are part of the UK Biobank, a prospective cohort study with deep phenotyping and genomics data for 500,000 individuals recruited between 2006 and 2010 (Allen et al., 2024). We only included individuals with quality-assured genotype data and information on at least one of five ACEs items from an online mental well-being questionnaire, resulting in a total of 163,704 individuals (Figure 1). The majority of the included individuals (91%) are of British ancestry.

### Definition of ACEs

Information on ACEs was derived from the Childhood Trauma Screener-5 items (CTS-5), which measures physical abuse, emotional abuse, sexual abuse, emotional neglect, and physical neglect using a Likert-type scale (Grabe et al., 2012). In the main analyses, ACEs were dichotomized as a binary variable, which was defined as ‘yes’ when the participants responded ‘Very often true’, ‘Often’, or ‘Sometimes true’ to one of the following three questions: (1) physical abuse: physically abused by family as a child; (2) emotional abuse: felt hated by family member as a child; and (3) sexual abuse: sexually molested as a child or responded ‘Never true’ or ‘Rarely true’ to one of the following two questions: (1) emotional neglect: felt loved as a child; (2) physical neglect: someone to take to doctor when needed as a child. The cut-offs for defining the existence of ACEs were selected based on prior studies (Jakubowski et al., 2023). The number of ACEs was calculated by summing these items.

### Measurement of psychopathological symptoms

Data on the lifetime incidence of psychopathological symptoms, except cognitive function, were collected via an online mental health self-assessment questionnaire sent out for completion in 2016 (Dutt et al., 2022). We included a total of 52 symptoms from six domains, including psychotic, mania, depression, anxiety, help-seeking, as well as self-harm or suicide. For the cognition domain, we employed results from three specific cognitive function tests on numeric memory, fluid intelligence, and prospective memory administered via a fully automated touchscreen questionnaire sent out for completion in 2014 (Fawns-Ritchie & Deary, 2020). While we considered seven domains and 55 individual symptoms, these only added up to 61 distinct phenotypes. As the help-seeking domain consisted of only one symptom, the domain and symptom were considered the same phenotype. We dichotomized each symptom into a binary variable with levels of ‘yes’ and ‘no’. Endorsement of the whole symptom domain was established if a participant reported ‘yes’ to at least one of the items (Supplementary Material 1). Information on the descriptions, data fields, sample sizes, and variable construction approaches of all items is presented in detail in Table S1 in Supplementary Material 2.

### Covariates

Besides sex and birth year, we included the following covariates: employment, income level, smoking status, education level, Index of Multiple Deprivation (IMD), and the first 10 genetically informative principal components (all measured at recruitment; Table S2 in Supplementary Material 2). IMD is an area-based measure of relative deprivation across each of the constituent nations of the UK, taking into account seven domains: income, employment, health deprivation, education, crime, barriers to housing and services, as well as living environment (Qi et al., 2022; Scopazzini et al., 2023).

### Polygenic risk scores

The target dataset for PRS calculation was the genotype data from the 163,704 included individuals, with approximately 850,000 variants; the quality control process has been described in detail elsewhere (Bycroft et al., 2018). The base datasets for calculating PRS-SCZ and PRS-BIP were the European subsets of recent genome-wide association studies for these two disorders from the Psychiatric Genomic Consortium, after excluding the individuals from the UK Biobank (Mullins et al., 2021; Trubetskoy et al., 2022). We adopted a PRS-PCA approach to generate the scores (Coombes, Ploner, Bergen, & Biernacka, 2020). PRSice 2 software (version 1.0.2) was employed to generate PRSs across 10 different thresholds (5e-8, 1e-6, 1e-4, 0.001, 0.01, 0.05, 0.1, 0.2, 0.5, 1) after clumping the SNPs at r^2^ < 0.1 within 250 kb (Choi & O’Reilly, 2019). After that, we applied principal component analysis (PCA) to scores at the 10 thresholds and derived the standardized first component as the PRS used for subsequent analyses. To make PRSs comparable with ACEs in scale, PRSs were dichotomized at the 75th percentile (Aas et al., 2023) (Supplementary Material 1).

### Statistical analysis

The flow of statistical analysis is illustrated in detail in Figure 1.

### Univariate effects of PRSs and ACEs on psychopathological symptoms

To investigate which psychopathological symptoms were significantly associated with PRS-SCZ, PRS-BIP, and ACEs, respectively, we fitted separate logistic regression models with each of the 55 individual psychopathological symptoms and 7 symptom domains as the outcome variable, and binary PRS-SCZ (high vs. low), PRS-BIP (high vs. low), and ACEs (yes vs. no) as the exposure variable, respectively, with all covariates adjusted for. We applied a Bonferroni-corrected significance level of *P* < 2.73 × 10^−4^ (0.05/ [61 × 3]). Only symptoms or domains demonstrating statistically significant associations for both PRS-SCZ/PRS-BIP and ACEs were included in the subsequent analysis (Supplementary Material 1).

### Joint effects of PRSs and ACEs on psychopathological symptoms

For psychopathological symptoms or domains showing significant associations with both PRS-SCZ/PRS-BIP and ACEs in the univariate models, we constructed logistic regression models incorporating both PRS-SCZ/PRS-BIP and ACEs as exposure variables, with all covariates adjusted for.

### Additive and multiplicative interactions between PRSs and ACEs on psychopathological symptoms

To estimate additive and multiplicative interactions between PRSs and ACEs, we first fitted logistic regression models incorporating both main effects from PRSs, ACEs, and their multiplicative interaction terms as exposures for all symptoms or domains that showed statistically significant univariate associations with both ACEs and the corresponding PRS, with all covariates adjusted for.

After that, to quantify additive interaction, we calculated the relative excess risk due to interaction (RERI) for each psychopathological symptom or domain considered, based on the ORs derived from these logistic regression models: OR _High PRS + ACEs_ − OR _Low PRS + ACEs_ − OR _High PRS + no ACEs_ + 1, which can also be interpretated as the difference between the combined risk of PRSs and ACEs (OR _High PRS + ACEs_) minus the excess risks of PRSs alone (OR _High PRS + no ACEs_ − 1) and excess risks of ACEs alone (OR _Low PRS + ACEs_ − 1). A RERI with a consistent sign with OR _High PRS + ACEs_ indicates the presence of an additive synergistic effect (combined effects greater than the sum of each effect alone), whereas a RERI with a discordant sign with OR _High PRS + ACEs_ indicates the presence of an additive antagonistic effect (combined effects smaller than the sum of each effect alone) (Knol, van der Tweel, Grobbee, Numans, & Geerlings, 2007; Knol & VanderWeele, 2012). The RERI confidence intervals were calculated with the delta method (Hosmer & Lemeshow, 1992).

Multiplicative interaction effects were shown on the log-odds ratio scale, corresponding to the regression coefficients for the multiplicative interaction term, which can also be represented as Log(OR_high PRS + ACEs_) − Log(OR_high PRS + no ACEs_) − Log(OR_low PRS + ACEs_) = Log [OR_high PRS + ACEs_*/*(OR_high PRS + no ACEs_ × OR_low PRS + ACEs_)]. A multiplicative interaction coefficient greater than zero indicates the presence of a multiplicative synergistic interaction effect, while a coefficient smaller than zero indicates the presence of a multiplicative antagonistic interaction. A multiplicative synergistic interaction indicates that the effects of ACEs are amplified among individuals with higher PRSs, whereas a multiplicative antagonistic interaction indicates that the effects of ACEs are ameliorated among individuals with higher PRSs, suggesting a ceiling or threshold effect. We applied a Bonferroni-corrected significance level to address the multiple comparison issues.

### Sensitivity analysis

As simply including the main effects of covariates may still bias the multiplicative interactions, we perform a sensitivity analysis to test the PRS × ACEs terms after further adjusting for covariate × PRS and covariate × ACEs terms (Keller, 2014).

Since the quality of PRSs is impaired when population ancestry differs between the discovery and target data, we repeated the univariate, joint, additive, and multiplicative interaction effect analyses after excluding individuals of non-British ancestry (N = 14,750).

To explore the dose-dependent associations and distinct association patterns for different types of ACEs, we tested the univariate and joint effects of the number or types of ACEs and PRSs on psychopathological symptom domains using the procedures described above.

All statistical analyses were performed with R version 4.2.3.

## Results

### Descriptive statistics

As shown in Table 1, of 163,704 included participants, 69,821 (43.7%) were male; the mean (SD) birth year was 1953 (8), corresponding to a median (IQR) age at recruitment of 57 (12). Compared with individuals without any ACEs, individuals reporting exposure to any ACEs showed generally higher PRSs for SCZ or BIP, lower income level, higher socioeconomic deprivation level, and greater risk of smoking. Proportions of reported psychopathological symptoms from all seven domains were consistently higher among individuals with exposure to any ACEs compared with those without.

**Table 1.** Descriptive statistics of included participants stratified by exposure to any ACEs

Variables	Overall (N=163,704)	Without any ACEs (N=128,511, 78.5%)	With any ACEs (N=35,193, 21.5%)
SCZ PRS (mean [SD])	−0.09 (0.90)	−0.13 (0.88)	0.02 (0.97)
BIP PRS (mean [SD])	−0.06 (0.95)	−0.09 (0.94)	0.03 (0.98)
Birth year (mean [SD])	1953 (8)	1952 (7)	1954 (8)
Sex = Male (%)	69,821 (42.7)	56,681 (44.1)	13,140 (37.3)
Total household Income (%)			
Less than *£* 18,000	18,742 (12.6)	13,309 (11.4)	5,433 (17.1)
*£* 18,000 to *£* 30,999	33,600 (22.7)	26,078 (22.4)	7,522 (23.6)
*£* 31,000 to *£* 51,999	43,641 (29.4)	34,457 (29.6)	9,184 (28.9)
*£* 52,000 to *£* 100,000	40,253 (27.2)	32,579 (28.0)	7,674 (24.1)
Greater than *£* 100,000	12,008 (8.1)	10,002 (8.6)	2,006 (6.3)
Employment = Employed (%)	100,227 (67.6)	78,104 (67.1)	22,123 (69.5)
Smoke = Yes (%)	67,541 (41.3)	50,360 (39.3)	17,181 (49.0)
Index of Multiple Deprivation (IMD, mean [SD])	14.26 (11.94)	13.72 (11.48)	16.21 (13.31)
Any psychotic symptoms (%)	5,166 (4.8)	3,190 (3.8)	1,976 (8.6)
Any mania symptoms (%)	28,719 (26.8)	19,637 (23.2)	9,082 (40.1)
Any depression symptoms (%)	52,446 (52.8)	39,848 (49.8)	12,598 (65.3)
Any anxiety symptoms (%)	20,215 (23.1)	15,640 (21.8)	4,575 (29.1)
Any help seeking (%)	42,219 (39.3)	29,759 (35.1)	12,460 (54.6)
Any self-harm symptoms (%)	35,101 (32.6)	23,371 (27.5)	11,730 (51.3)
Individuals with lower than average cognition level (%)	24,574 (41.7)	18,317 (39.8)	6,257 (48.6)
Recruitment year (mean [SD])	2009 (0.90)	2009 (0.90)	2009 (0.90)

ACEs, adverse childhood experiences; IMD, index of multiple deprivation; PRS, polygenic risk score.

*Note:* Recruitment year = year when first attending the assessment center. IMD is an area-based measure of relative deprivation across each of the constituent nations of the United Kingdom, taking into account seven domains, including income, employment, health deprivation, education, crime, barriers to housing and services, as well as living environment. Higher IMD indicates a higher socioeconomic deprivation level.

### Univariate and joint effects of PRSs and ACEs on psychopathological symptoms

ACEs shared 26 symptom associations with PRS-SCZ and 29 symptom associations with PRS-BIP in the univariate analyses (Supplementary Material 2, Table S3).

**Figure 2.**
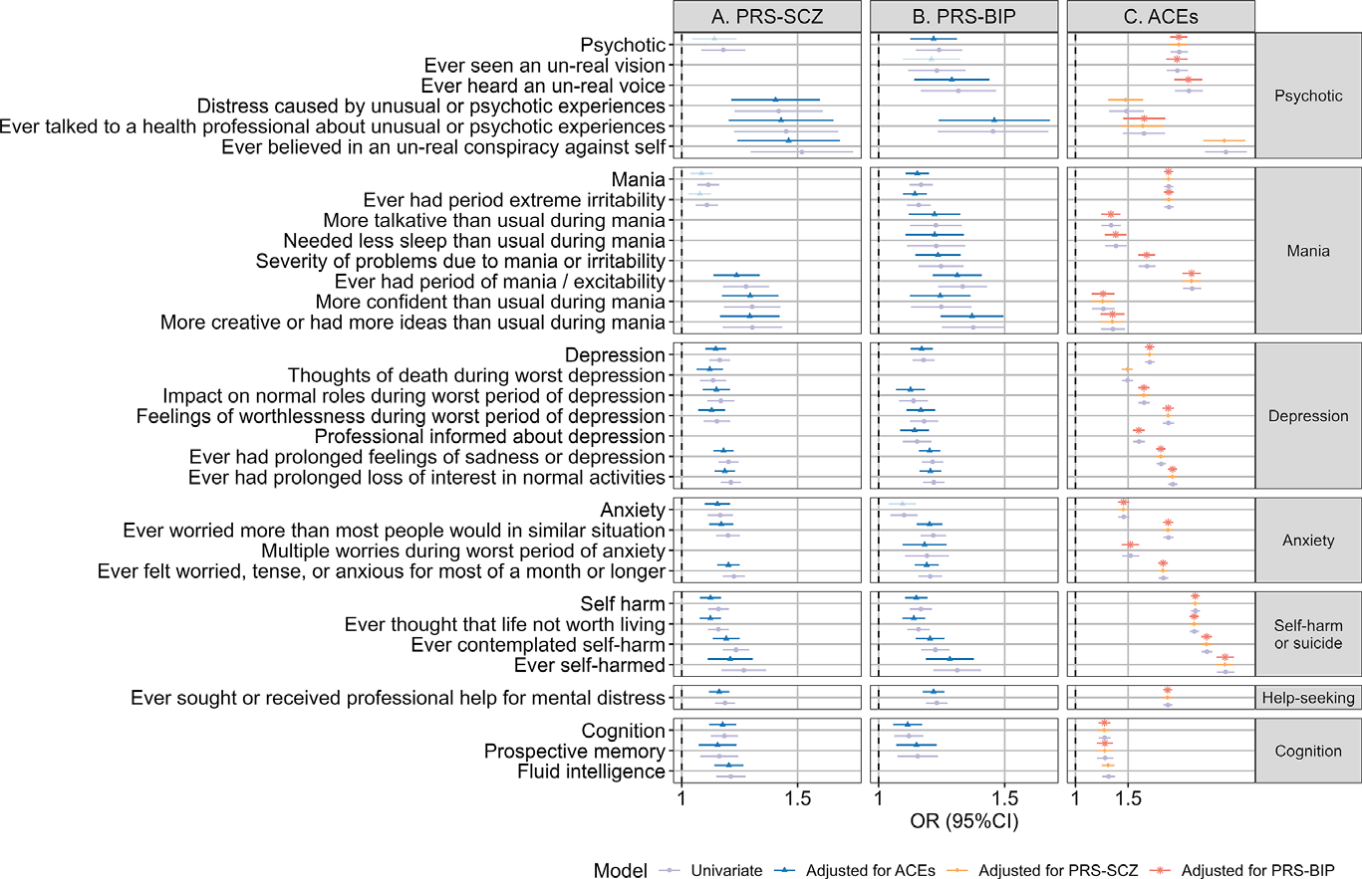
Joint effects of PRSs and ACEs on psychopathological symptoms. Odds ratios (95% confidence intervals) of PRS-SCZ (A) or PRS-BIP (B) and ACEs (C) on psychopathological symptoms in the joint and univariate models. The univariate estimates, joint estimates adjusting for ACEs, joint estimates adjusting for PRS-SCZ, and joint estimates adjusting for PRS-BIP are represented by purple circles, blue triangles, orange diamonds, and red asterisks, respectively. The error bars indicate confidence intervals. Joint estimates are derived from joint logistic regression models incorporating both PRS-SCZ or PRS-BIP and ACEs, after adjusting for covariates. Univariate estimates are derived from univariate logistic regression models for PRSs or ACEs, with covariates adjusted for. The x-axis indexes odd ratios, and the y-axis stands for psychopathological symptoms. The cut-off of high versus low PRS is set at the 75th percentile. Since we only included symptoms that showed statistically significant univariate associations with both ACEs and the corresponding PRS in the joint models, data are not shown for symptoms showing non-significant univariate associations with either ACEs or PRS-SCZ/PRS-BIP in the plot. Non-significant joint estimates for PRSs with ACEs adjusted for after Bonferroni correction (*P* < 2.73 × 10^−4^) were represented by transparent points. OR, odds ratio; 95%CI, 95% confidence intervals; PRS, polygenic risk scores; ACEs, adverse childhood experiences.

In the joint main effect models, PRS-SCZ, PRS-BIP, and exposure to any ACEs were still significantly associated with these psychopathological symptoms or domains that showed overlapping significant associations with PRSs and ACEs in the univariate analyses (Figure 2; Supplementary Material 2, Tables S4–S5). It is noteworthy that after adjusting for ACEs, the effects of PRSs on psychopathological symptoms showed minor decreases, whereas the effects of ACEs remained largely unchanged when PRSs were adjusted for. The strongest adjusted effect of PRS-SCZ after accounting for ACEs was observed on the item ‘ever believed in an un-real conspiracy against self’ (adjusted OR, 1.45; 95% CI, 1.21–1.74; R^2^ = 0.075), whereas the strongest adjusted effect of PRS-BIP after accounting for ACEs was for the item ‘ever talked to a health professional about unusual or psychotic experiences’ (adjusted OR, 1.45; 95% CI, 1.21–1.73; R^2^ = 0.074) from the psychotic domain. ACEs demonstrated the strongest association with the item ‘ever self-harmed’ after adjusting for PRS-SCZ or PRS-BIP (OR after adjusting for PRS-SCZ, 3.16, 95% CI, 2.96–3.38, R^2^ = 0.107; OR after adjusting for PRS-BIP, 3.17, 95% CI, 2.96–3.39, R^2^ = 0.108).

### Additive interactions of PRSs and ACEs on psychopathological symptoms

**Figure 3.**
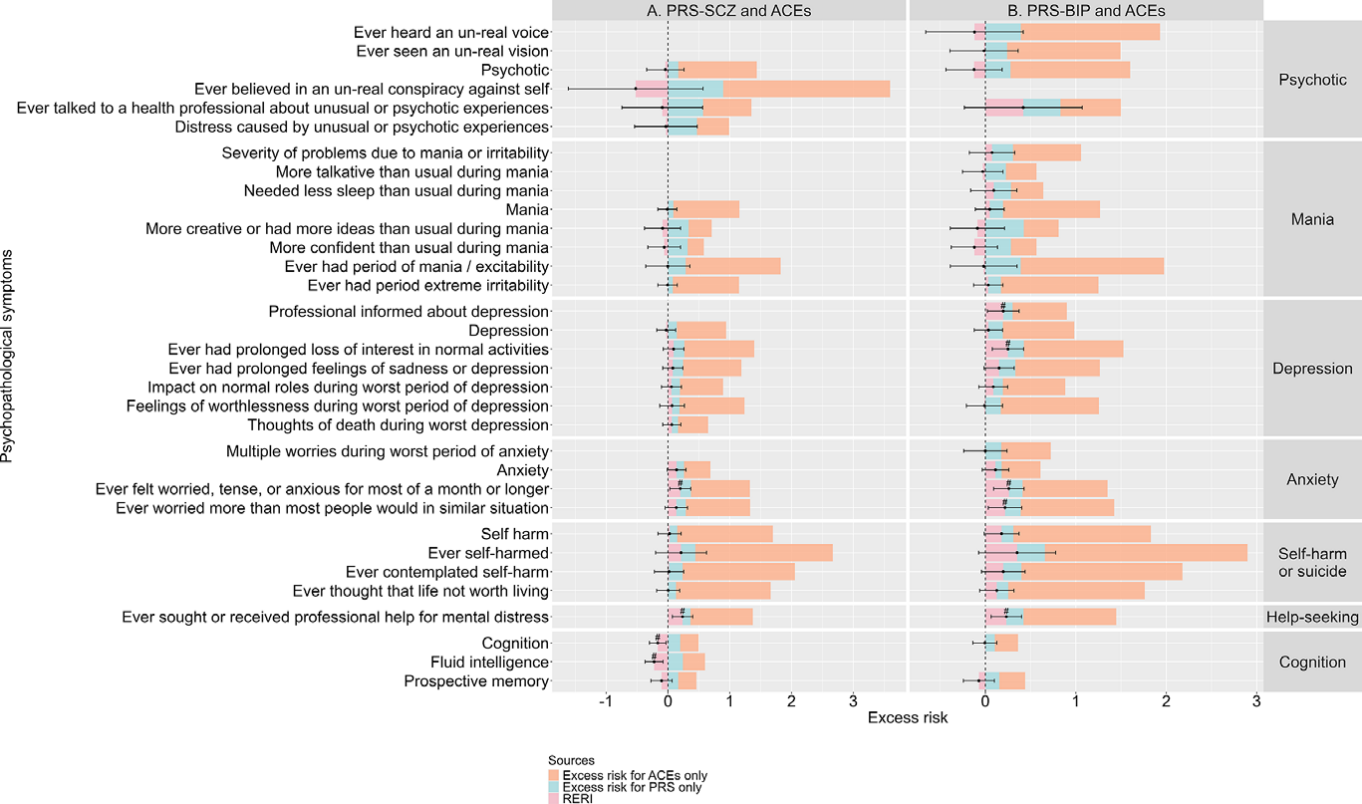
Additive interaction effects between PRSs and ACEs on psychopathological symptoms. Panels (a) and (b) show the additive interaction effects of PRS-SCZ (a) or PRS-BIP (b) and ACEs, by illustrating: (1) excess risk conferred by PRSs alone (blue bar); (2) excess risk conferred by ACEs alone (orange bar); (3) RERI (pink bar). The error bars represent the 95% confidence intervals of RERIs. The sum of these three components equals the combined risks of higher PRS and ACEs. The excess risks were calculated based on the ORs derived from logistic regression models incorporating main effects from PRSs (high vs. low), ACEs, and their multiplicative interaction term as exposures, with all covariates adjusted for. Excess risk of PRS alone was calculated as OR_high PRS + no ACEs_ − 1. Excess risk of ACEs alone was calculated as OR_low PRS + ACEs_ − 1. RERI was calculated as OR_high PRS + ACEs_ − OR_low PRS + ACEs_ − OR_high PRS + no ACEs_ + 1, which can be interpreted as the difference between the combined risk of PRSs and ACEs and the sum of risk for PRSs and ACEs alone. A positive RERI indicates a synergistic effect, whereas a negative RERI indicates an antagonistic effect. The x-axis indexes excess risk, and the y-axis displays psychopathological symptoms. The cut-off of the risk status of PRS was set at the 75^th^ percentile. Data are missing for symptoms with non-significant univariate associations with either ACEs or PRSs. # indicates nominal significance at *P* < 0.05. ACEs, adverse childhood experiences; 95%CI, 95% confidence intervals; OR, odds ratio; PRS, polygenic risk scores; RERI, relative excess risk due to interaction.

Statistically significant positive RERIs for PRS-SCZ and ACEs were observed for ‘ever felt worried, tense, or anxious for most of a month or longer’ (RERI, 0.20; 95%CI, 0.03–0.37; P, 0.021) from the anxiety domain and ‘ever sought or received professional help for mental distress’ from the help-seeking domain (RERI, 0.24; 95%CI, 0.07–0.4; P, 0.005), whereas statistically significant negative RERIs were detected for the whole cognition domain (RERI, −0.17; 95%CI, −0.3 to −0.03; P, 0.015) and fluid intelligence impairment (RERI, −0.23; 95%CI, −0.37 to −0.08; P, 0.002) from the cognition domain at *P* < 0.05 (Figure 3a and Supplementary Material 2, Table S6).

As indicated by the statistically significant positive RERIs (Figure 3b and Supplementary Material 2, Table S7), we found that combined effects of PRS-BIP and ACEs were greater than the sum of their effects alone on ‘professional informed about depression’ (RERI, 0.20; 95%CI, 0.02–0.37; P, 2.67 × 10^−2^) and ‘ever had prolonged loss of interest in normal activities’ (RERI, 0.25; 95%CI, 0.08–0.42; P, 4.70 × 10^−3^) from the depression domain; ‘ever felt worried, tense, or anxious for most of a month or longer’ (RERI, 0.26; 95%CI, 0.09–0.43; P, 2.47 × 10^−3^) and ‘ever worried more than most people would in similar situation’ (RERI, 0.22; 95%CI, 0.03–0.40; P, 2.14 × 10^−2^) from the anxiety domain; as well as ‘ever sought or received professional help for mental distress’ (RERI, 0.23; 95%CI, 0.06–0.40; P, 6.88 × 10^−3^) from the help-seeking domain at *P* < 0.05. However, none of these associations survived Bonferroni correction (*P <* 0.05/26 = 1.92 × 10^−3^ for PRS-SCZ and *P <* 0.05/29 = 1.72 × 10^−3^ for PRS-BIP).

### Multiplicative interaction effects of PRSs and ACEs on psychopathological symptoms

**Figure 4.**
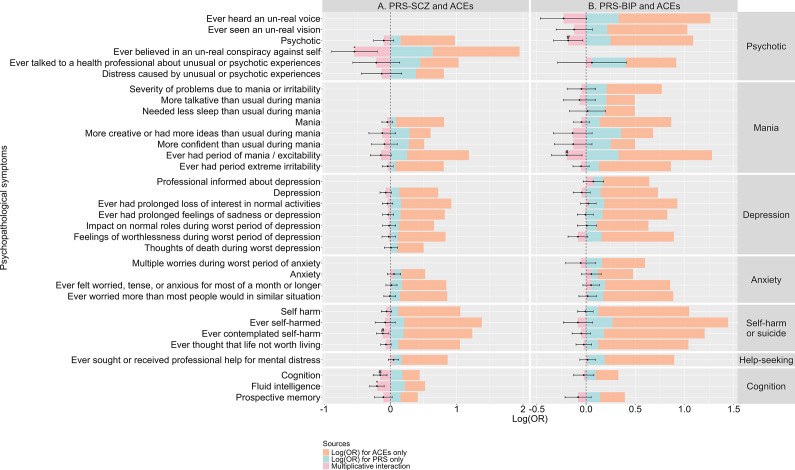
Multiplicative interaction effects between PRSs and ACEs on psychopathological symptoms. Panels (A) and (B) show the multiplicative interaction effects of PRS-SCZ (A) or PRS-BIP (B) and ACEs, by illustrating: (1) Log(OR) for PRSs alone (blue bar); (2) Log(OR) for ACEs alone (orange bar); (3) coefficient for multiplicative interaction term (pink bar). The error bars represent the 95% confidence intervals of multiplicative interaction. The excess risks were calculated based on the ORs derived from logistic regression models incorporating main effects from PRSs (high vs. low), ACEs, and their multiplicative interaction term as exposures, with all covariates adjusted for. Log(OR) for PRSs alone was calculated as Log(OR_high PRS + no ACEs_). Log(OR) for ACEs alone was calculated as Log(OR_low PRS + ACEs_). Multiplicative interaction was calculated as Log(OR_high PRS + ACEs_)−Log(OR_high PRS + no ACEs_)−Log(OR_low PRS + ACEs_). A positive multiplicative interaction indicates that the effects of ACEs are amplified among individuals with a higher PRS, whereas a negative multiplicative interaction indicates that the effects of ACEs are ameliorated among individuals with a higher PRS. The x-axis indexes log(OR), and the y-axis displays psychopathological symptoms. The cut-off of the risk status of PRS was set at the 75th percentile. Data are missing for symptoms with non-significant univariate associations with either PRSs or ACEs. # indicates nominal significance at *P < 0.05.* * indicates Bonferroni-corrected significance level (*P* < 0.05/26 for PRS-SCZ and ACEs; *P* < 0.05/29 for PRS-BIP and ACEs). ACEs, adverse childhood experiences; 95%CI, 95% confidence intervals; OR, odds ratio; PRS, polygenic risk scores.

Statistically significant multiplicative interactions for PRS-SCZ and ACEs were observed on ‘ever believed in an un-real conspiracy against self’ from the psychotic domain (coefficient, −0.543; SE, 0.175; P, 1.92 × 10^−3^), ‘ever contemplated self-harm’ from the self-harm or suicide domain (coefficient, −0.118; SE, 0.048; P, 1.47 × 10^−2^), the whole cognition domain (coefficient, −0.157; SE, 0.053; P, 3.18 × 10^−3^), and ‘fluid intelligence impairment’ from the cognition domain (coefficient, −0.204; SE, 0.055; P, 2.29 × 10^−4^). After Bonferroni correction, the effects on ‘ever believed in an un-real conspiracy against self’ and ‘fluid intelligence impairment’ remained statistically significant (*P <* 0.05/26 = 1.92 × 10^−3^; Figure 4A and Supplementary Material 2, Table S8).

For PRS-BIP and ACEs, statistically significant multiplicative interactions were observed for the whole psychotic domain (coefficient, −0.181; SE, 0.076; P, 1.72 × 10^−2^) and ‘ever had period of mania/excitability’ from the mania domain (coefficient, −0.195; SE, 0.079; P, 1.30 × 10^−2^) (Figure 4B; Supplementary Material 2, Table S9). However, these effects became non-significant after Bonferroni correction (*P <* 0.05/29 = 1.72 × 10^−3^).

### Sensitivity analyses

After further adjusting for covariates × ACEs and covariates × PRS interaction terms, the multiplicative interactions for PRSs and ACEs remained largely unchanged for all psychopathological symptoms (Supplementary Material 2, Table S10).

We observed similar findings from the analyses above after excluding non-British individuals.

We observed a dose-dependent relationship between the number of ACEs and increased risk across symptom domains in both univariate and joint models (Supplementary Material 2, Table S11–S13). Four different types of ACEs, physical abuse, emotional abuse, sexual abuse, and emotional neglect demonstrated statistically significant associations with all seven symptom domains in both univariate and joint models, with emotional abuse consistently showing the strongest effects on all domains except for cognition (Supplementary Material 2, Tables S14–S16).

## Discussion

We observe cross-domain and non-specific overlap between genetic liability to SCZ/BIP and ACEs in their associations with psychopathological symptoms. Additionally, ACEs and PRSs can independently predict these overlapping symptoms in the joint models. However, their interplay mechanisms vary across symptom domains and differ for the genetic liability to SCZ and BIP in terms of both strength and direction: multiplicative interactions were only detected on the psychotic, mania, self-harm or suicide, and cognitive domains, whereas additive interactions were observed on the depression, anxiety, and help-seeking and cognition domains. Both PRSs demonstrated additive interactions with ACEs on the anxiety and help-seeking domains, but multiplicative interactions on the psychotic domain. Specifically, PRS-SCZ showed a mix of additive and multiplicative interactions with ACEs on the cognition domain, whereas PRS-BIP and ACEs exhibited an additive interaction on the depression domain but a multiplicative interaction on the mania domain. Overall, this study provides novel insights into the heterogeneous gene–ACEs interaction mechanisms underlying psychosis, provides personalized guidance for psychological care after ACEs, and suggests the merit of a symptom-centered perspective in inspecting the gene–environment interactions on psychopathology.

Previous studies have confirmed independent effects of PRSs and ACEs on the risk of psychotic disorders, but our study generalized this finding to psychopathology more broadly, as well as to specific types and numbers of ACEs (Trotta et al., 2016). We found that ACEs and genetic liability are both relevant factors for psychopathology development, whereas ACEs might exert a more prominent influence compared with PRSs, consistent with the Stress–Vulnerability Model (Demke, 2022).

It is noteworthy that we only observed potential multiplicative interactions on the psychotic, mania, self-harm, and cognition domains, but not on the depression, anxiety, and help-seeking domains. Significant multiplicative interactions usually suggest that the two factors interplay via partially shared or interdependent biological pathways or mechanisms on the outcome, where the presence of one factor modifies or moderates the effect of the other at a cellular or molecular level (Nova, Fazia, & Bernardinelli, 2024). Therefore, it is not surprising that polygenic risks for SCZ and BIP demonstrate multiplicative interactions with ACEs only on the typical symptoms of SCZ or BIP. The absence of multiplicative interactions on more non-specific domains (i.e. depression, anxiety, and help-seeking) implies that genetic factors for SCZ or BIP and ACEs influence these domains via distinct pathways. However, the presence of additive interactions indicates amplified risks of depression, anxiety, and help-seeking behaviors associated with ACEs for individuals with higher genetic predisposition to SCZ or BIP, suggesting the essentiality of monitoring these symptoms among these individuals (Knol et al., 2007). The inconsistent interaction mechanisms across domains may also be attributable to differences in measurement scale or limited statistical power.

Both PRSs demonstrate strong additive synergistic interactions with ACEs on symptoms from anxiety and help-seeking domains, but multiplicative interactions on symptoms from the psychotic domain. Individuals genetically predisposed to SCZ or BIP are found to be more susceptible to generalized anxiety disorder and panic disorder, and per additional increase in number of ACEs exposed was associated with 54% excess risk of developing any anxiety disorders (Daníelsdóttir et al., 2024; Richards et al., 2019). Additionally, previous studies have revealed the association between genetic liability to SCZ and BIP and help-seeking behavior in psychosis patients. ACEs were also identified as a strong predictor for seeking help due to mental health issues (Karatekin, 2019; Scott et al., 2025). The multiplicative interactions with ACEs for both PRS-SCZ and PRS-BIP on positive symptoms (e.g. hallucinations, delusions) were also consistent with previous results (Alameda et al., 2024). These findings indicate that genetic risk for SCZ and BIP can interact with ACEs on anxiety, help-seeking, and psychotic experiences via similar mechanisms.

Interestingly, PRS-SCZ displayed a mixture of antagonistic additive and multiplicative interactions with ACEs, specifically on symptoms from the cognition domain. It is widely acknowledged that SCZ has a cognitive component, and genetic influences on SCZ might be partially mediated by cognition pathways (Toulopoulou et al., 2019). Additionally, exposure to ACEs was frequently identified to be associated with a lower late-life cognitive level (Patel & Oremus, 2023). Furthermore, some studies suggest that genetic and environmental factors might jointly influence the risk of psychosis through their shared effects on cognitive function (Park et al., 2024). Therefore, the antagonistic interaction direction can be a result of the ceiling effect: if an individual already has a high genetic burden for SCZ, the extra exposure to ACEs cannot diminish the cognition level to the same degree as it would in an individual with a lower genetic burden. These findings suggest the important role of cognition in mediating the interplay between the genetic risk of SCZ and ACEs on psychosis.

On the other hand, PRS-BIP and ACEs demonstrated a specific additive synergistic interaction on depression symptoms but a multiplicative antagonistic interaction on mania symptoms. It is noteworthy that depression and mania symptoms are both integral components of BIP, but their gene–ACEs interaction patterns are completely different in terms of both mechanisms and directions. These findings highlight the complexity and heterogeneity in gene–ACEs interplay mechanisms even within a single disorder.

This study has several limitations. First, information on ACEs and psychopathological symptoms was self-reported and collected retrospectively, which might lead to recall bias. However, previous studies indicate that retrospective measures of ACEs showed stronger associations with adulthood psychopathology compared with prospective measures, probably due to the fact that subjective appraisal and memory of ACEs can add to the effects conferred by the objective event alone on mental health outcomes (Baldwin, Reuben, Newbury, & Danese, 2019). These reveal the extra merits of adopting the retrospective measures. Second, selection bias might be involved, as not all participants in the UK Biobank completed the mental health questionnaire. Third, as the time for the onset of these psychopathological symptoms is not recorded, our analyses did not account for the effects of time. Fourth, as the participants included are mostly of European ancestry, the findings may not generalize to other ancestries. Finally, we cannot rule out the influences of PRS-ACEs correlations on the observed interaction effects (Supplementary Material 2, Table S17). However, previous studies suggested that gene–ACEs correlation effects are small enough that they will not seriously confound the interaction results (Woolway et al., 2022).

This study comprehensively investigates the joint contribution of genetic liability to SCZ or BIP and ACEs to individual psychopathological symptoms. We found that genetic factors and ACEs contributed jointly and independently to psychopathology in a transdiagnostic and non-specific manner. However, the mechanisms by which genetic factors and ACEs interplay may vary across symptoms and differ for SCZ and BIP. Our findings provide novel insights into the complex and heterogeneous gene–ACEs interplay mechanisms underlying psychosis and suggest the advantage of adopting a symptom-level perspective in dissecting the gene–environment interactions on psychopathology.

## Supporting information

10.1017/S0033291726104012.sm001 Lu et al. supplementary material Lu et al. supplementary material

## Supplementary material

The supplementary material for this article can be found at http://doi.org/10.1017/S0033291726104012.
