# Supplementary material for: Joint effects of childhood adversity and genetic risk for psychosis on psychopathology in the UK Biobank
Source: Psychol Med. 2026 Apr 7;56:e92. doi: 10.1017/S0033291726104012 (PMC13079213; doi:10.1017/S0033291726104012)
Supplement: Lu et al. supplementary material [file S0033291726104012sup001.pdf]

Supplementary Material 1:

Supplementary Methods

Measurement of psychopathological symptoms

Lifetime existence of most psychopathological symptoms were inquired about retrospectively with an online mental health self-assessment questionnaire sent out for completion in 2016 (Category 136) [1]. We included a total of 52 symptoms from six domains, which consisted of 7 items on psychotic experiences, 11 items on mania, 14 items on depression, 15 items on anxiety, 4 items on self harm or suicide and and 1 item on help seeking. As for the cognition domain, we employed results from three specific cognitive function tests on numeric memory (#4282), fluid intelligence (#20016), and prospective memory (#20018), administered via touch screen during the initial assessment [2]. For each symptom domain, there can be unconditional (asked to all participants) and conditional (asked only when the participant responded "yes" to some conditional items) items. We dichotomized each psychopathological symptom into a binary variable with levels of "yes" and "no". Endorsement of whole symptom domain was established if one participant reported "yes" to at least one of the items in the domain. Information on the descriptions, data fields, sample sizes and variable construction approaches of all psychopathological symptom items are presented in detail in Table S14.

Polygenic risk scores (PRSs)

The target dataset was the genotype data from 163,704 included individuals with approximately 850,000 variants, and the quality control process was described in detail elsewhere [3].The base datasets for calculating PRS-SCZ and PRS-BIP were the European subsets of recent genome-wide association studies for these two disorders from the Psychiatric Genomic Consortium, after excluding the

individuals from the UK Biobank [4, 5]. We adopted a PRS-PCA approach to generate the scores [6]. In the first step, we excluded all multiallelic and duplicated single nucleotides polymorphisms (SNPs) from the base GWAS datasets. After that, the genotype target dataset and base datasets were harmonized by aligning the effect alleles, excluding SNPs with mismatched alleles, and only retaining SNPs in both datasets. PRSice 2 software (version 1.0.2) was then employed to generate PRSs across 10 different thresholds (5e-8, 1e-6, 1e-4, 0.001,0.01, 0.05, 0.1, 0.2, 0.5, 1) after clumping the SNPs at $r^2 < 0.1$ within 250 kb. Finally, we applied principal component analysis (PCA) to scores at the 10 thresholds and derived the standardized first component as the PRS used for subsequent analyses. We performed PCA and standardization with R functions "princomp()" and "scale()", respectively.

Univariate effects of PRSs and ACEs on psychopathological symptoms

To investigate which psychopathological symptoms were significantly associated with PRS-SCZ, PRS-BIP and ACEs respectively, we fitted logistic regression model with each of the 55 individual psychopathological symptoms and 7 symptom domains as the outcome variable, and PRS-SCZ, PRS-BIP and ACEs as the exposure variable separately, with all covariates adjusted for. The ORs for PRSs on outcomes represented the risk estimates for high vs. low PRS, whereas the ORs for ACEs on outcomes indexed the increase in risk estimates for individuals with ACEs compared with those without. We applied a Bonferroni-corrected significance level of $P < 2.73 \times 10^{-4}$ (= 0.05/[61×3]) to address multiple comparisons. Only symptoms or domains demonstrating significant associations for both PRS-SCZ/PRS-BIP and ACEs were included in subsequent tests for joint, synergistic and multiplicative interaction effects.

References

1. Dutt, R.K., et al., *Mental health in the UK Biobank: A roadmap to self-report measures and neuroimaging correlates.* Hum Brain Mapp, 2022. **43**(2): p. 816-832.
2. Fawns-Ritchie, C. and I.J. Deary, *Reliability and validity of the UK Biobank cognitive tests.* PLoS One, 2020. **15**(4): p. e0231627.
3. Bycroft, C., et al., *The UK Biobank resource with deep phenotyping and genomic data.* Nature, 2018. **562**(7726): p. 203-209.
4. Trubetskoy, V., et al., *Mapping genomic loci implicates genes and synaptic biology in schizophrenia.* Nature, 2022. **604**(7906): p. 502-508.
5. Mullins, N., et al., *Genome-wide association study of more than 40,000 bipolar disorder cases provides new insights into the underlying biology.* Nat Genet, 2021. **53**(6): p. 817-829.
6. Coombes, B.J., et al., *A principal component approach to improve association testing with polygenic risk scores.* Genet Epidemiol, 2020. **44**(7): p. 676-686.
7. Knol, M.J., et al., *Estimating interaction on an additive scale between continuous determinants in a logistic regression model.* Int J Epidemiol, 2007. **36**(5): p. 1111-8.
8. Knol, M.J. and T.J. VanderWeele, *Recommendations for presenting analyses of effect modification and interaction.* Int J Epidemiol, 2012. **41**(2): p. 514-20.
9. Hosmer, D.W. and S. Lemeshow, *Confidence interval estimation of interaction.* Epidemiology, 1992. **3**(5): p. 452-6.

Joint effects of childhood adversity and genetic risk of psychosis on psychopathology in UK Biobank

Supplementary Material 2: Supplementary Tables

Table S1. Description of psychopathological symptom items.
Table S2. Description of covariates.
Table S3. Associations between PRS-SCZ, PRS-BIP or ACEs and psychopathological symptoms in the univariate analysis.
Table S4. Effects of PRS-SCZ and ACEs on psychopathological symptoms in the joint model analysis.
Table S5. Effects of PRS-BIP and ACEs on psychopathological symptoms in the joint model analysis.
Table S6. Additive interaction effects of PRS-SCZ and ACEs on psychopathological symptoms.
Table S7. Additive interaction effects of PRS-BIP and ACEs on psychopathological symptoms.
Table S8. Multiplicative interaction effects of PRS-SCZ and ACEs on psychopathological symptoms.
Table S9. Multiplicative interaction effects of PRS-BIP and ACEs on psychopathological symptoms.
Table S10. Multiplicative interaction effects of PRSs and ACEs on psychopathological symptoms after adjusting for the interaction terms between covariates and PRSs or ACEs.
Table S11. Effects of number of ACEs on psychopathological symptoms in the univariate models.
Table S12. Joint effects of PRS-SCZ and number of ACEs on psychopathological symptoms.
Table S13. Joint effects of PRS-BIP and number of ACEs on psychopathological symptoms.
Table S14. Effects of types of ACEs on psychopathological symptoms in the univariate models.
Table S15. Effects of PRS-SCZ and types of ACEs on psychopathological symptoms in the joint models.
Table S16. Effects of PRS-BIP and types of ACEs on psychopathological symptoms in the joint models.
Table S17. Associations between PRSs of SCZ or BIPs and ACEs, with all covariates adjusted for.

Table S1. Description of psychopathological symptom items.

Conditional = whether the item is asked to all participants (unconditional) or asked only to participants who responded "Yes" to some unconditional items (conditional).

Symptoms	Domain	Data field	Conditional	Coding = No	Coding = Yes	Sample size
Distress caused by unusual or psychotic experiences	Psychotic	20462	Yes; conditional on 20463, 20474, 20468	0,1	2,3,4	5057
Ever believed in an un-real conspiracy against self	Psychotic	20468	No	0	1	107579
Ever believed in un-real communications or signs	Psychotic	20474	No	0	1	107528
Ever heard an un-real voice	Psychotic	20463	No	0	1	107375
Ever prescribed a medication for unusual or psychotic experiences	Psychotic	20466	Yes; conditional on 20463, 20474, 20468	0	1	5124
Ever seen an un-real vision	Psychotic	20471	No	0	1	106708
Ever talked to a health professional about unusual or psychotic experiences	Psychotic	20477	No	0	1	5128
More talkative than usual during mania	Mania	20548	Yes; conditional on 20501, 20502	Without 1	1	19400
More restless than usual during mania	Mania	20548	Yes; conditional on 20501, 20502	Without 2	2	19400
Thoughts were racing during mania	Mania	20548	Yes; conditional on 20501, 20502	Without 3	3	19400
Needed less sleep than usual during mania	Mania	20548	Yes; conditional on 20501, 20502	Without 5	5	19400
More creative or had more ideas than usual during mania	Mania	20548	Yes; conditional on 20501, 20502	Without 6	6	19400
Easily distracted during mania	Mania	20548	Yes; conditional on 20501, 20502	Without 7	7	19400
More confident than usual during mania	Mania	20548	Yes; conditional on 20501, 20502	Without 8	8	19400
More active than usual during mania	Mania	20548	Yes; conditional on 20501, 20502	Without 9	9	19400
Ever had period extreme irritability	Mania	20502	No	0	1	104741
Ever had period of mania / excitability	Mania	20501	No	0	1	105559
Severity of problems due to mania or irritability	Mania	20493	Yes; conditional on 20501, 20502	0	1	24762

Impact on normal roles during worst period of depression	Depression	20440	Yes; conditional on 20441, 20446	0,1,2	3	60712
Ever had prolonged feelings of sadness or depression	Depression	20446	No	0	1	107604
Ever had prolonged loss of interest in normal activities	Depression	20441	No	0	1	107578
Depression possibly related to childbirth	Depression	20445	Yes; conditional on 20441, 20446	0	1	35591
Depression possibly related to stressful or traumatic event	Depression	20447	Yes; conditional on 20441, 20446	0	1	60631
Difficulty concentrating during worst depression	Depression	20435	Yes; conditional on 20441, 20446	0	1	53668
Feelings of tiredness during worst episode of depression	Depression	20449	Yes; conditional on 20441, 20446	0	1	54521
Feelings of worthlessness during worst period of depression	Depression	20450	Yes; conditional on 20441, 20446	0	1	56932
Professional informed about depression	Depression	20448	Yes; conditional on 20441, 20446	0	1	60582
Sleeping too much	Depression	20534	Yes; conditional on 20441, 20446	0	1	41546
Thoughts of death during worst depression	Depression	20437	Yes; conditional on 20441, 20446	0	1	57339
Trouble falling asleep	Depression	20533	Yes; conditional on 20441, 20446	0	1	41546
Waking too early	Depression	20535	Yes; conditional on 20441, 20446	0	1	41546
Weight change during worst episode of depression	Depression	20536	Yes; conditional on 20441, 20446	0	1,2,3	51676
Difficulty concentrating during worst period of anxiety	Anxiety	20419	Yes; conditional on 20425, 20420	0	1	31765
Difficulty stopping worrying during worst period of anxiety	Anxiety	20541	Yes; conditional on 20425, 20420	0	1	32404
Easily tired during worst period of anxiety	Anxiety	20429	Yes; conditional on 20425, 20420	0	1	31005

Ever felt worried, tense, or anxious for most of a month or longer	Anxiety	20421	No	0	1	101359
Ever worried more than most people would in similar situation	Anxiety	20425	No	0	1	90607
Frequent trouble falling or staying asleep during worst period of anxiety	Anxiety	20427	Yes; conditional on 20425, 20420	0	1	32453
Impact on normal roles during worst period of anxiety	Anxiety	20418	Yes; conditional on 20425, 20420	0	1	33262
Keyed up or on edge during worst period of anxiety	Anxiety	20423	Yes; conditional on 20425, 20420	0	1	31743
More irritable than usual during worst period of anxiety	Anxiety	20422	Yes; conditional on 20425, 20420	0	1	30460
Multiple worries during worst period of anxiety	Anxiety	20540	Yes; conditional on 20425, 20420	0	1	31913
Professional informed about anxiety	Anxiety	20428	Yes; conditional on 20425, 20420	0	1	33137
Restless during period of worst anxiety	Anxiety	20426	Yes; conditional on 20425, 20420	0	1	30882
Stronger worrying (than other people) during period of worst anxiety	Anxiety	20542	Yes; conditional on 20425, 20420	0	1	22878
Tense, sore, or aching muscles during worst period of anxiety	Anxiety	20417	Yes; conditional on 20425, 20420	0	1	30379
Worried most days during period of worst anxiety	Anxiety	20538	Yes; conditional on 20425, 20420	0	1	32145
Ever thought that life not worth living	Self-harm and suicide	20479	No	0	1,2	107221
Ever contemplated self-harm	Self-harm and suicide	20485	No	0	1,2	107452
Ever self-harmed	Self-harm and suicide	20480	No	0	1	107535
Ever attempted suicide	Self-harm and suicide	20483	Yes; conditional on 20480	0	1	4602

Ever sought or received professional help for mental distress	Help-seeking	20499	No	0	1	107531
Prospective memory impairment	Cognition	20018	No	0,2	1	58867
Numeric memory impairment	Cognition	4282	No	> 6.95097	<=6.95097	17673
Fluid intelligence impairment	Cognition	20016	No	> 6.664528	<=6.664528	58427

Table S2. Description of covariates.

Covariates	Data field	Conditional	Type of variable	Coding	Sample size
Birth year	34	No	Continuous	/	163704
Sex	31	No	Binary	0 = Female, 1 = Male	163704
Income	738	No	Categorical	0 = Less than 18000; 1 = 18000 to 20999; 2 = 31000 to 51999; 3 = 52000 to 100000; 4 = Greater tahn 100000	148244
Employment	6142	No	Binary	1 = "In paid employment or self-employed"; 0 = others	148249
Smoking	20116	No	Binary	1 = "Current"|"Previous"; 0 = "Never"	163347
Enrollment year	53	No	Continuous	/	163704
PC1	22009	No	Continuous	/	163704
PC2	22009	No	Continuous	/	163704
PC3	22009	No	Continuous	/	163704
PC4	22009	No	Continuous	/	163704
PC5	22009	No	Continuous	/	163704
PC6	22009	No	Continuous	/	163704
PC7	22009	No	Continuous	/	163704
PC8	22009	No	Continuous	/	163704
PC9	22009	No	Continuous	/	163704
PC10	22009	No	Continuous	/	163704
Deprivation score	26410, 26426, 26427	No	Continuous	/	163704
Education	26414, 26431,26421	No	Continuous	/	163704

Table S3. Associations between PRS-SCZ, PRS-BIP or ACEs and psychopathological symptoms in the univariate analysis.
OR_all = odds ratio among all individuals; CI_all = 95% confidence intervals among all individuals; R2_all = Nagelkerke R2 among all individuals; P_all = P value among all individuals; OR_onlyBritish = odds ratio after excluding non-British individuals; CI_onlyBritish = 95% confidence intervals after excluding non-British individuals; R2_onlyBritish = Nagelkerke R2 after excluding non-British individuals; P_onlyBritish = P value after excluding non-British individuals; PRS = polygenic risk score; ACEs = adverse childhood experiences; Significance = whether the association is significant after Bonferroni correction at P < 2.73 × 10-4. Effects were derived from univariate models with only PRS-SCZ or PRS-BIP or ACEs, with all the covariates adjusted for.

Domain	Symptoms	Exposure	OR_all	CI_all	P_all	R2_all	significance_all	OR_onlyBritish	CI_onlyBritish	P_onlyBritish	R2_onlyBritish	significance_onlyBritish
**Univariate associations with PRS-SCZ**												
**Psychotic**												
Psychotic	Ever believed in an un-real conspiracy against self	PRS-SCZ	1.52	1.27 - 1.82	4.12E-06	0.050	TRUE	1.53	1.26 - 1.87	1.91E-05	0.045	TRUE
Psychotic	Ever prescribed a medication for unusual or psychotic experiences	PRS-SCZ	1.45	1.15 - 1.83	1.63E-03	0.075		1.42	1.1 - 1.83	7.48E-03	0.072	
Psychotic	Ever talked to a health professional about unusual or psychotic experiences	PRS-SCZ	1.44	1.2 - 1.73	8.31E-05	0.059	TRUE	1.45	1.18 - 1.77	3.16E-04	0.056	TRUE
Psychotic	Distress caused by unusual or psychotic experiences	PRS-SCZ	1.40	1.2 - 1.64	1.67E-05	0.043	TRUE	1.41	1.19 - 1.67	7.43E-05	0.041	TRUE
Psychotic	Ever believed in un-real communications or signs	PRS-SCZ	1.31	1.07 - 1.59	7.51E-03	0.028		1.36	1.1 - 1.69	4.42E-03	0.025	
Psychotic	Psychotic	PRS-SCZ	1.16	1.07 - 1.25	2.00E-04	0.020	TRUE	1.14	1.04 - 1.24	3.03E-03	0.021	
Psychotic	Ever heard an un-real voice	PRS-SCZ	1.16	1.02 - 1.31	2.16E-02	0.026		1.12	0.97 - 1.28	1.28E-01	0.028	
Psychotic	Ever seen an un-real vision	PRS-SCZ	1.11	1.01 - 1.22	3.48E-02	0.019		1.07	0.97 - 1.19	1.88E-01	0.019	
**Mania**												
Mania	More confident than usual during mania	PRS-SCZ	1.28	1.16 - 1.41	1.02E-06	0.017	TRUE	1.25	1.12 - 1.4	5.42E-05	0.016	TRUE
Mania	More creative or had more ideas than usual during mania	PRS-SCZ	1.28	1.15 - 1.42	3.25E-06	0.021	TRUE	1.25	1.12 - 1.41	1.31E-04	0.021	TRUE
Mania	Ever had period of mania / excitability	PRS-SCZ	1.25	1.16 - 1.36	4.46E-08	0.040	TRUE	1.26	1.15 - 1.38	2.78E-07	0.036	TRUE
Mania	More talkative than usual during mania	PRS-SCZ	1.17	1.07 - 1.27	4.13E-04	0.009		1.18	1.08 - 1.3	4.42E-04	0.009	TRUE
Mania	Needed less sleep than usual during mania	PRS-SCZ	1.17	1.07 - 1.29	1.07E-03	0.009		1.18	1.06 - 1.31	2.28E-03	0.009	
Mania	More active than usual during mania	PRS-SCZ	1.16	1.06 - 1.26	8.13E-04	0.006		1.16	1.06 - 1.28	1.37E-03	0.008	
Mania	Severity of problems due to mania or irritability	PRS-SCZ	1.11	1.03 - 1.19	7.03E-03	0.018		1.12	1.04 - 1.22	5.09E-03	0.017	
Mania	Thoughts were racing during mania	PRS-SCZ	1.11	1.03 - 1.2	8.70E-03	0.017		1.14	1.04 - 1.24	3.16E-03	0.017	
Mania	Mania	PRS-SCZ	1.10	1.06 - 1.14	1.95E-06	0.047	TRUE	1.09	1.04 - 1.14	6.42E-05	0.046	TRUE
Mania	Ever had period extreme irritability	PRS-SCZ	1.09	1.05 - 1.14	9.34E-06	0.048	TRUE	1.09	1.04 - 1.13	1.42E-04	0.047	TRUE
Mania	Easily distracted during mania	PRS-SCZ	0.97	0.9 - 1.05	4.41E-01	0.038		0.98	0.9 - 1.07	6.30E-01	0.041	
Mania	More restless than usual during mania	PRS-SCZ	0.94	0.87 - 1.02	1.20E-01	0.006		0.95	0.87 - 1.03	2.35E-01	0.005	
**Depression**												
Depression	Ever had prolonged loss of interest in normal activities	PRS-SCZ	1.19	1.15 - 1.23	1.33E-21	0.076	TRUE	1.20	1.15 - 1.24	6.04E-20	0.076	TRUE
Depression	Ever had prolonged feelings of sadness or depression	PRS-SCZ	1.18	1.14 - 1.22	4.91E-20	0.072	TRUE	1.18	1.14 - 1.23	9.75E-18	0.073	TRUE
Depression	Impact on normal roles during worst period of depression	PRS-SCZ	1.15	1.09 - 1.2	1.52E-08	0.020	TRUE	1.14	1.08 - 1.2	7.88E-07	0.019	TRUE
Depression	Depression	PRS-SCZ	1.14	1.1 - 1.18	5.96E-13	0.053	TRUE	1.14	1.1 - 1.19	4.33E-11	0.053	TRUE

Depression	Feelings of worthlessness during worst period of depression	PRS-SCZ	1.13	1.08 - 1.18	2.03E-07	0.053	TRUE	1.13	1.08 - 1.19	1.21E-06	0.055	TRUE
Depression	Thoughts of death during worst depression	PRS-SCZ	1.12	1.07 - 1.17	2.77E-06	0.024	TRUE	1.12	1.06 - 1.18	1.16E-05	0.024	TRUE
Depression	Sleeping too much	PRS-SCZ	1.08	1.01 - 1.15	2.16E-02	0.038		1.11	1.03 - 1.19	4.52E-03	0.036	
Depression	Professional informed about depression	PRS-SCZ	1.07	1.02 - 1.12	4.98E-03	0.041		1.06	1 - 1.11	3.89E-02	0.038	
Depression	Difficulty concentrating during worst depression	PRS-SCZ	1.05	0.99 - 1.11	9.67E-02	0.037		1.05	0.98 - 1.12	1.37E-01	0.038	
Depression	Waking too early	PRS-SCZ	1.05	0.99 - 1.12	1.10E-01	0.003		1.07	1 - 1.14	5.30E-02	0.003	
Depression	Depression possibly related to stressful or traumatic event	PRS-SCZ	1.04	0.99 - 1.09	1.33E-01	0.043		1.02	0.97 - 1.08	4.19E-01	0.044	
Depression	Weight change during worst episode of depression	PRS-SCZ	1.01	0.96 - 1.07	6.03E-01	0.097		1.02	0.97 - 1.08	3.82E-01	0.099	
Depression	Depression possibly related to childbirth	PRS-SCZ	1.00	0.91 - 1.09	9.95E-01	0.007		1.04	0.94 - 1.14	4.43E-01	0.007	
Depression	Feelings of tiredness during worst episode of depression	PRS-SCZ	0.99	0.93 - 1.05	8.14E-01	0.046		1.00	0.94 - 1.07	9.87E-01	0.045	
Depression	Trouble falling asleep	PRS-SCZ	0.94	0.88 - 1	3.59E-02	0.007		0.93	0.87 - 0.99	3.11E-02	0.007	
**Anxiety**												
Anxiety	Ever felt worried, tense, or anxious for most of a month or longer	PRS-SCZ	1.20	1.15 - 1.25	2.99E-20	0.030	TRUE	1.19	1.14 - 1.24	1.98E-16	0.029	TRUE
Anxiety	Ever worried more than most people would in similar situation	PRS-SCZ	1.18	1.13 - 1.23	3.03E-14	0.045	TRUE	1.17	1.12 - 1.23	3.70E-12	0.045	TRUE
Anxiety	Anxiety	PRS-SCZ	1.14	1.09 - 1.2	2.27E-09	0.013	TRUE	1.14	1.08 - 1.19	1.83E-07	0.012	TRUE
Anxiety	Tense, sore, or aching muscles during worst period of anxiety	PRS-SCZ	1.10	1.03 - 1.17	3.68E-03	0.059		1.09	1.02 - 1.17	1.35E-02	0.061	
Anxiety	Professional informed about anxiety	PRS-SCZ	1.09	1.02 - 1.15	7.85E-03	0.023		1.10	1.03 - 1.18	4.74E-03	0.019	
Anxiety	Impact on normal roles during worst period of anxiety	PRS-SCZ	1.09	0.97 - 1.21	1.35E-01	0.012		1.04	0.92 - 1.17	5.67E-01	0.011	
Anxiety	Difficulty concentrating during worst period of anxiety	PRS-SCZ	1.08	1 - 1.16	3.60E-02	0.039		1.08	1 - 1.17	5.85E-02	0.040	
Anxiety	Multiple worries during worst period of anxiety	PRS-SCZ	1.07	1 - 1.15	5.17E-02	0.033		1.08	1 - 1.16	5.73E-02	0.035	
Anxiety	Restless during period of worst anxiety	PRS-SCZ	1.05	0.99 - 1.12	1.07E-01	0.023		1.05	0.98 - 1.13	1.49E-01	0.022	
Anxiety	Easily tired during worst period of anxiety	PRS-SCZ	1.05	0.98 - 1.12	1.51E-01	0.030		1.05	0.98 - 1.13	1.78E-01	0.032	
Anxiety	Frequent trouble falling or staying asleep during worst period of anxiety	PRS-SCZ	1.01	0.94 - 1.1	7.60E-01	0.029		1.00	0.92 - 1.09	9.72E-01	0.029	
Anxiety	Stronger worrying (than other people) during period of worst anxiety	PRS-SCZ	1.00	0.91 - 1.09	9.20E-01	0.037		0.98	0.88 - 1.08	6.68E-01	0.038	
Anxiety	Worried most days during period of worst anxiety	PRS-SCZ	1.00	0.91 - 1.09	9.21E-01	0.017		1.01	0.91 - 1.12	9.13E-01	0.016	
Anxiety	More irritable than usual during worst period of anxiety	PRS-SCZ	0.99	0.93 - 1.06	8.25E-01	0.032		1.00	0.93 - 1.08	9.49E-01	0.034	
Anxiety	Keyed up or on edge during worst period of anxiety	PRS-SCZ	0.97	0.9 - 1.04	4.00E-01	0.012		0.95	0.88 - 1.03	2.34E-01	0.005	

Anxiety	Difficulty stopping worrying during worst period of anxiety	PRS-SCZ	0.97	0.86 - 1.11	6.95E-01	0.018		0.98	0.85 - 1.14	8.32E-01	0.014	
**Help seeking**												
Help-seeking	Ever sought or received professional help for mental distress	PRS-SCZ	1.16	1.12 - 1.21	3.59E-17	0.075	TRUE	1.17	1.13 - 1.22	1.03E-15	0.071	TRUE
**Self-harm or suicide**												
Self-harm or suicide	Ever self-harmed	PRS-SCZ	1.24	1.15 - 1.34	4.58E-08	0.068	TRUE	1.24	1.14 - 1.35	7.78E-07	0.069	TRUE
Self-harm or suicide	Ever contemplated self-harm	PRS-SCZ	1.21	1.15 - 1.27	7.33E-16	0.063	TRUE	1.24	1.17 - 1.3	1.53E-16	0.064	TRUE
Self-harm or suicide	Self harm	PRS-SCZ	1.14	1.1 - 1.18	3.15E-12	0.056	TRUE	1.16	1.11 - 1.2	7.48E-13	0.055	TRUE
Self-harm or suicide	Ever thought that life not worth living	PRS-SCZ	1.14	1.1 - 1.18	7.16E-12	0.052	TRUE	1.16	1.11 - 1.2	1.31E-12	0.051	TRUE
Self-harm or suicide	Ever attempted suicide	PRS-SCZ	1.14	0.97 - 1.33	1.09E-01	0.058		1.09	0.92 - 1.3	3.03E-01	0.058	
**Cognition**												
Cognition	Fluid intelligence impairment	PRS-SCZ	1.19	1.13 - 1.25	3.36E-11	0.084	TRUE	1.20	1.13 - 1.27	2.12E-10	0.047	TRUE
Cognition	Cognition	PRS-SCZ	1.16	1.11 - 1.22	6.34E-10	0.083	TRUE	1.18	1.12 - 1.24	8.63E-10	0.056	TRUE
Cognition	Prospective memory impairment	PRS-SCZ	1.14	1.07 - 1.22	9.92E-05	0.061	TRUE	1.14	1.06 - 1.23	5.87E-04	0.023	TRUE
Cognition	Numeric memory impairment	PRS-SCZ	1.03	0.94 - 1.12	5.67E-01	0.026		1.02	0.93 - 1.13	6.28E-01	0.023	
**Univariate associations with PRS-BIP**												
**Psychotic**												
Psychotic	Ever prescribed a medication for unusual or psychotic experiences	PRS-BIP	1.56	1.25 - 1.96	1.07E-04	0.077	TRUE	1.55	1.21 - 1.99	4.53E-04	0.075	TRUE
Psychotic	Ever talked to a health professional about unusual or psychotic experiences	PRS-BIP	1.44	1.21 - 1.73	5.05E-05	0.059	TRUE	1.43	1.17 - 1.74	3.62E-04	0.056	TRUE
Psychotic	Ever believed in an un-real conspiracy against self	PRS-BIP	1.38	1.15 - 1.65	4.47E-04	0.048		1.43	1.17 - 1.74	3.85E-04	0.044	TRUE
Psychotic	Ever heard an un-real voice	PRS-BIP	1.29	1.15 - 1.46	3.07E-05	0.027	TRUE	1.23	1.07 - 1.4	3.05E-03	0.028	
Psychotic	Ever believed in un-real communications or signs	PRS-BIP	1.24	1.02 - 1.5	3.32E-02	0.027		1.17	0.94 - 1.46	1.57E-01	0.024	
Psychotic	Distress caused by unusual or psychotic experiences	PRS-BIP	1.23	1.06 - 1.43	7.36E-03	0.039		1.34	1.14 - 1.58	5.04E-04	0.040	TRUE
Psychotic	Psychotic	PRS-BIP	1.21	1.13 - 1.31	3.44E-07	0.021	TRUE	1.20	1.1 - 1.3	1.69E-05	0.021	TRUE
Psychotic	Ever seen an un-real vision	PRS-BIP	1.21	1.1 - 1.32	6.86E-05	0.020	TRUE	1.19	1.07 - 1.31	8.85E-04	0.020	TRUE
**Mania**												
Mania	More creative or had more ideas than usual during mania	PRS-BIP	1.36	1.23 - 1.5	3.39E-09	0.023	TRUE	1.38	1.23 - 1.54	1.45E-08	0.023	TRUE
Mania	Ever had period of mania / excitability	PRS-BIP	1.31	1.21 - 1.42	1.53E-11	0.040	TRUE	1.31	1.2 - 1.43	1.43E-09	0.037	TRUE
Mania	Severity of problems due to mania or irritability	PRS-BIP	1.22	1.14 - 1.31	5.03E-08	0.020	TRUE	1.24	1.15 - 1.34	7.73E-08	0.018	TRUE
Mania	More confident than usual during mania	PRS-BIP	1.22	1.11 - 1.35	4.84E-05	0.016	TRUE	1.23	1.1 - 1.37	1.85E-04	0.016	TRUE
Mania	More talkative than usual during mania	PRS-BIP	1.20	1.11 - 1.31	1.31E-05	0.010	TRUE	1.20	1.1 - 1.32	7.42E-05	0.010	TRUE

Mania	Needed less sleep than usual during mania	PRS-BIP	1.20	1.1 - 1.32	1.05E-04	0.009	TRUE	1.20	1.08 - 1.33	5.31E-04	0.009	TRUE
Mania	Mania	PRS-BIP	1.15	1.1 - 1.19	6.26E-13	0.047	TRUE	1.17	1.12 - 1.21	1.12E-13	0.047	TRUE
Mania	More active than usual during mania	PRS-BIP	1.15	1.06 - 1.25	7.22E-04	0.006		1.18	1.07 - 1.29	4.82E-04	0.008	TRUE
Mania	Ever had period extreme irritability	PRS-BIP	1.14	1.1 - 1.18	2.58E-11	0.048	TRUE	1.16	1.11 - 1.21	2.66E-12	0.048	TRUE
Mania	Easily distracted during mania	PRS-BIP	1.10	1.02 - 1.19	9.92E-03	0.038		1.09	1.01 - 1.19	3.75E-02	0.041	
Mania	More restless than usual during mania	PRS-BIP	1.01	0.94 - 1.09	7.71E-01	0.006		1.03	0.95 - 1.12	4.95E-01	0.005	
Mania	Thoughts were racing during mania	PRS-BIP	1.01	0.94 - 1.09	7.83E-01	0.016		1.00	0.92 - 1.08	9.20E-01	0.017	
**Depression**												
Depression	Ever had prolonged loss of interest in normal activities	PRS-BIP	1.19	1.15 - 1.24	6.70E-24	0.076	TRUE	1.22	1.18 - 1.27	1.02E-25	0.077	TRUE
Depression	Ever had prolonged feelings of sadness or depression	PRS-BIP	1.19	1.15 - 1.23	2.80E-23	0.073	TRUE	1.22	1.17 - 1.26	1.11E-24	0.073	TRUE
Depression	Depression	PRS-BIP	1.16	1.12 - 1.2	1.25E-15	0.053	TRUE	1.17	1.13 - 1.22	2.95E-16	0.053	TRUE
Depression	Feelings of worthlessness during worst period of depression	PRS-BIP	1.16	1.11 - 1.21	2.87E-10	0.053	TRUE	1.18	1.12 - 1.24	1.08E-10	0.055	TRUE
Depression	Professional informed about depression	PRS-BIP	1.13	1.08 - 1.19	1.45E-07	0.041	TRUE	1.12	1.06 - 1.17	1.77E-05	0.039	TRUE
Depression	Impact on normal roles during worst period of depression	PRS-BIP	1.12	1.07 - 1.17	2.26E-06	0.019	TRUE	1.13	1.07 - 1.19	2.84E-06	0.019	TRUE
Depression	Thoughts of death during worst depression	PRS-BIP	1.09	1.04 - 1.14	2.90E-04	0.024		1.09	1.04 - 1.14	8.22E-04	0.024	TRUE
Depression	Difficulty concentrating during worst depression	PRS-BIP	1.09	1.03 - 1.16	2.44E-03	0.037		1.08	1.02 - 1.15	1.44E-02	0.038	
Depression	Sleeping too much	PRS-BIP	1.09	1.02 - 1.16	9.51E-03	0.038		1.11	1.03 - 1.19	4.21E-03	0.036	
Depression	Weight change during worst episode of depression	PRS-BIP	1.06	1.01 - 1.12	1.83E-02	0.097		1.07	1.01 - 1.13	1.44E-02	0.100	
Depression	Feelings of tiredness during worst episode of depression	PRS-BIP	1.04	0.98 - 1.11	1.59E-01	0.046		1.04	0.97 - 1.11	2.44E-01	0.045	
Depression	Waking too early	PRS-BIP	1.04	0.98 - 1.1	2.47E-01	0.003		1.03	0.96 - 1.1	4.18E-01	0.003	
Depression	Depression possibly related to childbirth	PRS-BIP	1.02	0.94 - 1.12	6.29E-01	0.007		1.03	0.93 - 1.13	5.75E-01	0.007	
Depression	Depression possibly related to stressful or traumatic event	PRS-BIP	0.97	0.92 - 1.02	2.23E-01	0.043		0.97	0.92 - 1.03	2.92E-01	0.044	
Depression	Trouble falling asleep	PRS-BIP	0.96	0.9 - 1.02	1.48E-01	0.007		0.96	0.9 - 1.03	2.60E-01	0.007	
**Anxiety**												
Anxiety	Ever worried more than most people would in similar situation	PRS-BIP	1.19	1.14 - 1.24	3.59E-17	0.046	TRUE	1.20	1.15 - 1.25	1.15E-15	0.045	TRUE
Anxiety	Ever felt worried, tense, or anxious for most of a month or longer	PRS-BIP	1.18	1.14 - 1.23	1.34E-17	0.030	TRUE	1.20	1.15 - 1.25	1.97E-17	0.029	TRUE
Anxiety	Multiple worries during worst period of anxiety	PRS-BIP	1.17	1.09 - 1.25	1.49E-05	0.034	TRUE	1.18	1.09 - 1.27	2.88E-05	0.036	TRUE
Anxiety	Impact on normal roles during worst period of anxiety	PRS-BIP	1.14	1.02 - 1.27	1.70E-02	0.012		1.12	0.99 - 1.26	6.87E-02	0.011	
Anxiety	Stronger worrying (than other people) during period of worst anxiety	PRS-BIP	1.11	1.01 - 1.23	2.47E-02	0.037		1.10	0.99 - 1.22	7.53E-02	0.039	
Anxiety	Difficulty stopping worrying during worst period of anxiety	PRS-BIP	1.11	0.97 - 1.26	1.20E-01	0.018		1.09	0.95 - 1.26	2.26E-01	0.014	

Anxiety	Anxiety	PRS-BIP	1.09	1.04 - 1.13	2.38E-04	0.012	TRUE	1.10	1.05 - 1.15	1.22E-04	0.011	TRUE
Anxiety	Difficulty concentrating during worst period of anxiety	PRS-BIP	1.09	1.02 - 1.17	1.50E-02	0.040		1.11	1.02 - 1.19	1.06E-02	0.041	
Anxiety	Professional informed about anxiety	PRS-BIP	1.08	1.02 - 1.15	8.91E-03	0.023		1.07	1 - 1.14	4.04E-02	0.018	
Anxiety	Tense, sore, or aching muscles during worst period of anxiety	PRS-BIP	1.08	1.02 - 1.15	1.47E-02	0.058		1.08	1.01 - 1.16	2.82E-02	0.061	
Anxiety	More irritable than usual during worst period of anxiety	PRS-BIP	1.08	1.01 - 1.16	2.05E-02	0.033		1.09	1.02 - 1.18	1.84E-02	0.035	
Anxiety	Easily tired during worst period of anxiety	PRS-BIP	1.07	1 - 1.14	5.32E-02	0.031		1.08	1 - 1.16	5.15E-02	0.032	
Anxiety	Keyed up or on edge during worst period of anxiety	PRS-BIP	1.04	0.97 - 1.12	2.85E-01	0.012		1.04	0.96 - 1.13	2.96E-01	0.005	
Anxiety	Restless during period of worst anxiety	PRS-BIP	1.03	0.97 - 1.1	2.84E-01	0.023		1.03	0.96 - 1.1	4.59E-01	0.022	
Anxiety	Frequent trouble falling or staying asleep during worst period of anxiety	PRS-BIP	1.02	0.94 - 1.1	6.25E-01	0.029		0.99	0.91 - 1.08	8.33E-01	0.029	
Anxiety	Worried most days during period of worst anxiety	PRS-BIP	0.96	0.88 - 1.05	4.01E-01	0.018		0.97	0.88 - 1.07	5.71E-01	0.016	
**Help-seeking**												
Help-seeking	Ever sought or received professional help for mental distress	PRS-BIP	1.21	1.16 - 1.25	1.98E-26	0.075	TRUE	1.22	1.17 - 1.26	6.33E-25	0.072	TRUE
**Self-harm or suicide**												
Self-harm or suicide	Ever self-harmed	PRS-BIP	1.29	1.19 - 1.39	8.21E-11	0.069	TRUE	1.34	1.23 - 1.46	4.55E-12	0.070	TRUE
Self-harm or suicide	Ever attempted suicide	PRS-BIP	1.22	1.05 - 1.42	9.64E-03	0.059		1.20	1.01 - 1.42	3.32E-02	0.060	
Self-harm or suicide	Ever contemplated self-harm	PRS-BIP	1.20	1.15 - 1.26	3.18E-15	0.063	TRUE	1.24	1.18 - 1.3	3.07E-17	0.064	TRUE
Self-harm or suicide	Self harm	PRS-BIP	1.15	1.11 - 1.19	6.41E-14	0.056	TRUE	1.17	1.13 - 1.22	1.92E-15	0.055	TRUE
Self-harm or suicide	Ever thought that life not worth living	PRS-BIP	1.14	1.1 - 1.18	2.48E-12	0.052	TRUE	1.16	1.12 - 1.21	4.89E-14	0.052	TRUE
**Cognition**												
Cognition	Prospective memory impairment	PRS-BIP	1.13	1.06 - 1.21	1.44E-04	0.061	TRUE	1.14	1.06 - 1.23	4.70E-04	0.023	TRUE
Cognition	Cognition	PRS-BIP	1.10	1.05 - 1.15	4.28E-05	0.082	TRUE	1.09	1.04 - 1.15	6.29E-04	0.056	TRUE
Cognition	Fluid intelligence impairment	PRS-BIP	1.10	1.04 - 1.15	3.57E-04	0.083		1.08	1.02 - 1.14	7.74E-03	0.045	
Cognition	Numeric memory impairment	PRS-BIP	0.99	0.91 - 1.08	7.81E-01	0.026		0.99	0.9 - 1.09	8.51E-01	0.022	
**Univariate associations with ACEs**												
**Psychotic**												
Psychotic	Ever believed in an un-real conspiracy against self	ACEs	3.19	2.71 - 3.74	2.39E-45	0.073	TRUE	3.41	2.85 - 4.06	3.12E-42	0.071	TRUE
Psychotic	Ever believed in un-real communications or signs	ACEs	2.45	2.07 - 2.91	1.09E-24	0.041	TRUE	2.49	2.06 - 3	2.32E-21	0.038	TRUE
Psychotic	Ever heard an un-real voice	ACEs	2.40	2.15 - 2.67	2.13E-57	0.043	TRUE	2.41	2.14 - 2.71	1.16E-48	0.044	TRUE
Psychotic	Psychotic	ACEs	2.22	2.08 - 2.38	5.48E-123	0.038	TRUE	2.28	2.12 - 2.45	3.99E-111	0.040	TRUE
Psychotic	Ever seen an un-real vision	ACEs	2.19	2.02 - 2.38	5.25E-79	0.034	TRUE	2.24	2.05 - 2.45	7.33E-72	0.036	TRUE

Psychotic	Ever talked to a health professional about unusual or psychotic experiences	ACEs	1.70	1.44 - 1.99	1.59E-10	0.068	TRUE	1.60	1.34 - 1.91	1.87E-07	0.062	TRUE
Psychotic	Distress caused by unusual or psychotic experiences	ACEs	1.48	1.3 - 1.69	8.15E-09	0.048	TRUE	1.46	1.27 - 1.69	2.85E-07	0.045	TRUE
Psychotic	Ever prescribed a medication for unusual or psychotic experiences	ACEs	1.32	1.07 - 1.63	1.00E-02	0.074		1.28	1.02 - 1.62	3.25E-02	0.071	
**Mania**												
Mania	Ever had period of mania / excitability	ACEs	2.46	2.29 - 2.64	6.11E-139	0.061	TRUE	2.44	2.26 - 2.64	7.95E-113	0.057	TRUE
Mania	Ever had period extreme irritability	ACEs	2.06	1.98 - 2.13	0.00E+00	0.072	TRUE	2.07	1.99 - 2.15	1.89E-291	0.071	TRUE
Mania	Mania	ACEs	2.05	1.98 - 2.12	0.00E+00	0.071	TRUE	2.06	1.98 - 2.14	3.81E-296	0.070	TRUE
Mania	Severity of problems due to mania or irritability	ACEs	1.74	1.63 - 1.85	2.43E-62	0.036	TRUE	1.76	1.64 - 1.88	4.22E-55	0.035	TRUE
Mania	Needed less sleep than usual during mania	ACEs	1.37	1.26 - 1.48	2.21E-13	0.013	TRUE	1.36	1.24 - 1.49	3.15E-11	0.013	TRUE
Mania	More active than usual during mania	ACEs	1.35	1.25 - 1.45	1.21E-15	0.011	TRUE	1.34	1.24 - 1.46	8.86E-13	0.012	TRUE
Mania	More creative or had more ideas than usual during mania	ACEs	1.33	1.22 - 1.46	7.57E-10	0.023	TRUE	1.32	1.19 - 1.47	8.45E-08	0.023	TRUE
Mania	More talkative than usual during mania	ACEs	1.32	1.22 - 1.42	3.18E-13	0.013	TRUE	1.31	1.21 - 1.42	6.36E-11	0.013	TRUE
Mania	Thoughts were racing during mania	ACEs	1.27	1.19 - 1.36	1.47E-12	0.020	TRUE	1.28	1.19 - 1.38	2.87E-11	0.021	TRUE
Mania	More confident than usual during mania	ACEs	1.24	1.14 - 1.35	1.41E-06	0.017	TRUE	1.21	1.1 - 1.34	9.73E-05	0.016	TRUE
Mania	More restless than usual during mania	ACEs	1.15	1.07 - 1.23	6.18E-05	0.007	TRUE	1.14	1.06 - 1.23	5.13E-04	0.006	
Mania	Easily distracted during mania	ACEs	1.14	1.07 - 1.22	8.92E-05	0.039	TRUE	1.11	1.04 - 1.2	4.03E-03	0.042	
**Depression**												
Depression	Ever had prolonged loss of interest in normal activities	ACEs	2.12	2.05 - 2.19	0.00E+00	0.101	TRUE	2.12	2.04 - 2.2	0.00E+00	0.101	TRUE
Depression	Feelings of worthlessness during worst period of depression	ACEs	2.05	1.96 - 2.14	8.10E-225	0.081	TRUE	2.04	1.94 - 2.14	2.80E-187	0.081	TRUE
Depression	Ever had prolonged feelings of sadness or depression	ACEs	1.94	1.87 - 2.01	5.81E-293	0.091	TRUE	1.96	1.89 - 2.04	8.53E-254	0.091	TRUE
Depression	Depression	ACEs	1.77	1.71 - 1.84	1.48E-201	0.067	TRUE	1.80	1.72 - 1.87	2.54E-175	0.067	TRUE
Depression	Impact on normal roles during worst period of depression	ACEs	1.70	1.63 - 1.77	3.55E-130	0.035	TRUE	1.70	1.62 - 1.78	3.23E-111	0.034	TRUE
Depression	Professional informed about depression	ACEs	1.63	1.56 - 1.71	5.56E-101	0.053	TRUE	1.60	1.53 - 1.68	1.71E-79	0.050	TRUE
Depression	Difficulty concentrating during worst depression	ACEs	1.57	1.49 - 1.67	3.04E-54	0.046	TRUE	1.59	1.49 - 1.69	1.92E-46	0.047	TRUE
Depression	Thoughts of death during worst depression	ACEs	1.49	1.43 - 1.56	3.05E-76	0.033	TRUE	1.49	1.42 - 1.56	7.22E-64	0.033	TRUE
Depression	Weight change during worst episode of depression	ACEs	1.47	1.4 - 1.54	6.83E-57	0.104	TRUE	1.46	1.38 - 1.53	1.45E-46	0.106	TRUE
Depression	Feelings of tiredness during worst episode of depression	ACEs	1.43	1.35 - 1.52	7.22E-32	0.051	TRUE	1.48	1.38 - 1.58	1.71E-31	0.051	TRUE
Depression	Sleeping too much	ACEs	1.40	1.33 - 1.49	5.88E-31	0.044	TRUE	1.39	1.3 - 1.48	9.74E-25	0.041	TRUE

Depression	Depression possibly related to childbirth	ACEs	1.21	1.12 - 1.31	3.24E-06	0.009	TRUE	1.21	1.11 - 1.32	1.01E-05	0.008	TRUE
Depression	Waking too early	ACEs	1.09	1.03 - 1.15	3.85E-03	0.003		1.09	1.02 - 1.16	6.72E-03	0.003	
Depression	Trouble falling asleep	ACEs	1.06	1 - 1.12	5.19E-02	0.007		1.07	1.01 - 1.14	3.31E-02	0.007	
Depression	Depression possibly related to stressful or traumatic event	ACEs	0.86	0.82 - 0.9	9.94E-11	0.044	TRUE	0.85	0.81 - 0.9	4.40E-10	0.045	TRUE
**Anxiety**												
Anxiety	Ever worried more than most people would in similar situation	ACEs	2.05	1.97 - 2.13	4.36E-286	0.068	TRUE	2.04	1.96 - 2.13	7.54E-240	0.067	TRUE
Anxiety	Ever felt worried, tense, or anxious for most of a month or longer	ACEs	1.97	1.9 - 2.04	8.17E-291	0.050	TRUE	1.96	1.89 - 2.04	3.61E-244	0.049	TRUE
Anxiety	Impact on normal roles during worst period of anxiety	ACEs	1.61	1.45 - 1.79	2.10E-18	0.018	TRUE	1.61	1.43 - 1.8	2.37E-15	0.018	TRUE
Anxiety	Tense, sore, or aching muscles during worst period of anxiety	ACEs	1.56	1.47 - 1.65	4.50E-52	0.070	TRUE	1.57	1.48 - 1.68	1.88E-46	0.073	TRUE
Anxiety	Multiple worries during worst period of anxiety	ACEs	1.53	1.43 - 1.64	4.05E-36	0.042	TRUE	1.58	1.47 - 1.7	1.11E-34	0.045	TRUE
Anxiety	Anxiety	ACEs	1.45	1.39 - 1.52	9.53E-63	0.018	TRUE	1.42	1.36 - 1.49	3.29E-47	0.016	TRUE
Anxiety	Professional informed about anxiety	ACEs	1.41	1.33 - 1.49	5.99E-34	0.030	TRUE	1.40	1.32 - 1.49	4.51E-28	0.025	TRUE
Anxiety	Difficulty concentrating during worst period of anxiety	ACEs	1.40	1.31 - 1.5	4.78E-24	0.045	TRUE	1.43	1.33 - 1.54	2.45E-22	0.046	TRUE
Anxiety	Keyed up or on edge during worst period of anxiety	ACEs	1.40	1.31 - 1.5	1.21E-21	0.018	TRUE	1.41	1.31 - 1.53	5.50E-19	0.011	TRUE
Anxiety	More irritable than usual during worst period of anxiety	ACEs	1.39	1.31 - 1.49	1.74E-24	0.038	TRUE	1.42	1.32 - 1.52	1.05E-22	0.041	TRUE
Anxiety	Restless during period of worst anxiety	ACEs	1.39	1.32 - 1.48	1.73E-30	0.030	TRUE	1.41	1.32 - 1.5	2.68E-27	0.030	TRUE
Anxiety	Stronger worrying (than other people) during period of worst anxiety	ACEs	1.39	1.27 - 1.52	1.99E-13	0.042	TRUE	1.42	1.29 - 1.57	1.00E-12	0.044	TRUE
Anxiety	Difficulty stopping worrying during worst period of anxiety	ACEs	1.36	1.21 - 1.54	6.23E-07	0.020	TRUE	1.38	1.2 - 1.58	4.41E-06	0.016	TRUE
Anxiety	Easily tired during worst period of anxiety	ACEs	1.34	1.26 - 1.42	7.51E-20	0.035	TRUE	1.37	1.28 - 1.47	1.53E-19	0.037	TRUE
Anxiety	Worried most days during period of worst anxiety	ACEs	1.33	1.21 - 1.45	4.79E-10	0.020	TRUE	1.33	1.2 - 1.47	1.46E-08	0.019	TRUE
Anxiety	Frequent trouble falling or staying asleep during worst period of anxiety	ACEs	1.31	1.22 - 1.41	6.48E-13	0.033	TRUE	1.33	1.22 - 1.44	9.90E-12	0.033	TRUE
**Help seeking**												
Help-seeking	Ever sought or received professional help for mental distress	ACEs	2.04	1.97 - 2.11	0.00E+00	0.098	TRUE	2.04	1.97 - 2.12	0.00E+00	0.094	TRUE
**Self-harm or suicide**												
Self-harm or suicide	Ever self-harmed	ACEs	3.18	2.97 - 3.4	9.97E-252	0.107	TRUE	3.19	2.97 - 3.44	1.10E-212	0.107	TRUE
Self-harm or suicide	Ever contemplated self-harm	ACEs	2.75	2.64 - 2.87	0.00E+00	0.104	TRUE	2.78	2.66 - 2.91	0.00E+00	0.105	TRUE
Self-harm or suicide	Self harm	ACEs	2.52	2.44 - 2.61	0.00E+00	0.097	TRUE	2.54	2.45 - 2.64	0.00E+00	0.095	TRUE
Self-harm or suicide	Ever thought that life not worth living	ACEs	2.50	2.42 - 2.59	0.00E+00	0.092	TRUE	2.52	2.43 - 2.62	0.00E+00	0.091	TRUE

Self-harm or suicide	Ever attempted suicide	ACEs	1.49	1.3 - 1.7	5.36E-09	0.068	TRUE	1.54	1.33 - 1.78	7.10E-09	0.071	TRUE
**Cognition**												
Cognition	Fluid intelligence impairment	ACEs	1.29	1.23 - 1.36	6.40E-25	0.086	TRUE	1.34	1.27 - 1.41	1.96E-26	0.049	TRUE
Cognition	Prospective memory impairment	ACEs	1.26	1.18 - 1.34	4.61E-13	0.062	TRUE	1.32	1.23 - 1.41	2.70E-14	0.026	TRUE
Cognition	Cognition	ACEs	1.25	1.2 - 1.31	5.77E-22	0.084	TRUE	1.30	1.23 - 1.36	5.57E-24	0.059	TRUE
Cognition	Numeric memory impairment	ACEs	1.20	1.11 - 1.31	1.81E-05	0.028	TRUE	1.23	1.13 - 1.35	5.70E-06	0.025	TRUE

Table S4. Effects of PRS-SCZ and ACEs on psychopathological symptoms in the joint model analysis.

OR_all = odds ratio among all individuals; CI_all = 95% confidence intervals among all individuals; R2_all = Nagelkerke R2 among all individuals; P_all = P value among all individuals; OR_onlyBritish = odds ratio after excluding non-British individuals; CI_onlyBritish = 95% confidence intervals after excluding non-British individuals; R2_onlyBritish = Nagelkerke R2 after excluding non-British individuals; P_onlyBritish = P value after excluding non-British individuals; SCZ = schizophrenia; PRS = polygenic risk score; ACEs = adverse childhood experiences. Effects were derived from joint models incorporating both PRS-SCZ and ACEs, with all the covariates adjusted for.

Domain	Symptoms	Exposure	OR_all	CI_all	P_all	R2_all	OR_onlyBritish	CI_onlyBritish	P_onlyBritish	R2_onlyBritish
**Psychotic**										
Psychotic	Distress caused by unusual or psychotic experiences	ACEs	1.47	1.29 - 1.68	1.59E-08	0.053	1.45	1.26 - 1.68	5.25E-07	0.050
Psychotic	Distress caused by unusual or psychotic experiences	PRS-SCZ	1.39	1.19 - 1.62	3.32E-05	0.053	1.39	1.17 - 1.65	1.39E-04	0.050
Psychotic	Ever believed in an un-real conspiracy against self	ACEs	3.15	2.68 - 3.7	2.31E-44	0.075	3.36	2.82 - 4.01	2.32E-41	0.073
Psychotic	Ever believed in an un-real conspiracy against self	PRS-SCZ	1.45	1.21 - 1.74	4.38E-05	0.075	1.46	1.2 - 1.78	1.55E-04	0.073
Psychotic	Ever talked to a health professional about unusual or psychotic experiences	ACEs	1.68	1.43 - 1.97	3.53E-10	0.073	1.58	1.33 - 1.89	3.70E-07	0.067
Psychotic	Ever talked to a health professional about unusual or psychotic experiences	PRS-SCZ	1.42	1.18 - 1.7	1.97E-04	0.073	1.42	1.16 - 1.74	6.58E-04	0.067
Psychotic	Psychotic	ACEs	2.22	2.07 - 2.37	1.03E-121	0.039				
Psychotic	Psychotic	PRS-SCZ	1.12	1.04 - 1.21	3.65E-03	0.039				
**Mania**										
Mania	Ever had period extreme irritability	ACEs	2.05	1.98 - 2.13	0.00E+00	0.072	2.07	1.99 - 2.15	8.93E-290	0.071
Mania	Ever had period extreme irritability	PRS-SCZ	1.07	1.02 - 1.11	1.62E-03	0.072	1.06	1.02 - 1.11	8.02E-03	0.071
Mania	Ever had period of mania / excitability	ACEs	2.44	2.28 - 2.62	5.36E-137	0.062	2.43	2.25 - 2.62	3.32E-111	0.058
Mania	Ever had period of mania / excitability	PRS-SCZ	1.21	1.12 - 1.31	3.46E-06	0.062	1.22	1.12 - 1.34	1.04E-05	0.058
Mania	Mania	ACEs	2.05	1.98 - 2.12	0.00E+00	0.071	2.05	1.98 - 2.13	1.98E-294	0.070
Mania	Mania	PRS-SCZ	1.07	1.03 - 1.11	4.56E-04	0.071	1.06	1.02 - 1.11	3.93E-03	0.070
Mania	More confident than usual during mania	PRS-SCZ	1.27	1.15 - 1.4	2.07E-06	0.019	1.25	1.12 - 1.39	8.48E-05	0.018
Mania	More confident than usual during mania	ACEs	1.23	1.13 - 1.35	2.81E-06	0.019	1.21	1.09 - 1.33	1.51E-04	0.018
Mania	More creative or had more ideas than usual during mania	ACEs	1.33	1.21 - 1.46	1.68E-09	0.025	1.32	1.19 - 1.46	1.48E-07	0.025
Mania	More creative or had more ideas than usual during mania	PRS-SCZ	1.27	1.14 - 1.41	7.47E-06	0.025	1.24	1.11 - 1.4	2.36E-04	0.025
**Depression**										
Depression	Depression	ACEs	1.77	1.7 - 1.84	2.63E-199	0.067	1.79	1.72 - 1.87	1.54E-173	0.067
Depression	Depression	PRS-SCZ	1.13	1.09 - 1.17	1.50E-10	0.067	1.13	1.08 - 1.17	3.55E-09	0.067
Depression	Ever had prolonged feelings of sadness or depression	ACEs	1.93	1.86 - 2	2.77E-289	0.092	1.95	1.88 - 2.03	9.64E-251	0.092
Depression	Ever had prolonged feelings of sadness or depression	PRS-SCZ	1.16	1.12 - 1.2	4.44E-16	0.092	1.16	1.12 - 1.21	1.95E-14	0.092
Depression	Ever had prolonged loss of interest in normal activities	ACEs	2.11	2.04 - 2.18	0.00E+00	0.102	2.11	2.03 - 2.19	0.00E+00	0.102
Depression	Ever had prolonged loss of interest in normal activities	PRS-SCZ	1.16	1.12 - 1.2	1.31E-16	0.102	1.17	1.13 - 1.22	9.62E-16	0.102
Depression	Feelings of worthlessness during worst period of depression	ACEs	2.04	1.95 - 2.13	3.43E-223	0.081	2.03	1.94 - 2.13	6.32E-186	0.082
Depression	Feelings of worthlessness during worst period of depression	PRS-SCZ	1.11	1.06 - 1.16	1.24E-05	0.081	1.11	1.06 - 1.17	3.78E-05	0.082
Depression	Impact on normal roles during worst period of depression	ACEs	1.69	1.62 - 1.77	1.31E-128	0.036	1.69	1.62 - 1.77	5.62E-110	0.035
Depression	Impact on normal roles during worst period of depression	PRS-SCZ	1.13	1.08 - 1.18	6.18E-07	0.036	1.12	1.06 - 1.18	1.52E-05	0.035
Depression	Thoughts of death during worst depression	ACEs	1.49	1.43 - 1.55	2.91E-75	0.034	1.49	1.42 - 1.56	4.59E-63	0.034
Depression	Thoughts of death during worst depression	PRS-SCZ	1.1	1.05 - 1.15	3.04E-05	0.034	1.11	1.05 - 1.16	8.36E-05	0.034
**Anxiety**										
Anxiety	Anxiety	ACEs	1.45	1.38 - 1.51	1.37E-61	0.018	1.42	1.35 - 1.49	2.27E-46	0.016
Anxiety	Anxiety	PRS-SCZ	1.13	1.08 - 1.18	3.25E-08	0.018	1.13	1.07 - 1.18	1.27E-06	0.016
Anxiety	Ever felt worried, tense, or anxious for most of a month or longer	ACEs	1.96	1.89 - 2.03	8.77E-287	0.052	1.96	1.88 - 2.03	5.04E-241	0.050
Anxiety	Ever felt worried, tense, or anxious for most of a month or longer	PRS-SCZ	1.18	1.13 - 1.22	3.11E-16	0.052	1.17	1.12 - 1.22	2.71E-13	0.050
Anxiety	Ever worried more than most people would in similar situation	ACEs	2.04	1.96 - 2.12	2.03E-282	0.069	2.03	1.95 - 2.12	6.36E-237	0.068
Anxiety	Ever worried more than most people would in similar situation	PRS-SCZ	1.15	1.1 - 1.2	1.41E-10	0.069	1.15	1.1 - 1.2	3.13E-09	0.068
**Help seeking**										

Help-seeking	Ever sought or received professional help for mental distress	ACEs	2.03	1.96 - 2.1	0.00E+00	0.099	2.03	1.96 - 2.11	0.00E+00	0.095
Help-seeking	Ever sought or received professional help for mental distress	PRS-SCZ	1.14	1.1 - 1.18	5.72E-13	0.099	1.15	1.1 - 1.19	3.39E-12	0.095
**Self-harm or suicide**										
Self-harm or suicide	Ever contemplated self-harm	ACEs	2.74	2.63 - 2.86	0.00E+00	0.105	2.77	2.65 - 2.89	0.00E+00	0.106
Self-harm or suicide	Ever contemplated self-harm	PRS-SCZ	1.17	1.11 - 1.22	8.37E-11	0.105	1.2	1.14 - 1.26	6.19E-12	0.106
Self-harm or suicide	Ever self-harmed	ACEs	3.16	2.96 - 3.38	5.42E-249	0.107	3.18	2.95 - 3.42	1.89E-210	0.107
Self-harm or suicide	Ever self-harmed	PRS-SCZ	1.18	1.1 - 1.28	2.45E-05	0.107	1.18	1.09 - 1.29	1.31E-04	0.107
Self-harm or suicide	Ever thought that life not worth living	ACEs	2.49	2.41 - 2.58	0.00E+00	0.093	2.51	2.42 - 2.61	0.00E+00	0.092
Self-harm or suicide	Ever thought that life not worth living	PRS-SCZ	1.11	1.06 - 1.15	1.42E-07	0.093	1.13	1.08 - 1.17	1.17E-08	0.092
Self-harm or suicide	Self harm	ACEs	2.51	2.43 - 2.6	0.00E+00	0.097	2.53	2.44 - 2.63	0.00E+00	0.096
Self-harm or suicide	Self harm	PRS-SCZ	1.11	1.07 - 1.15	9.18E-08	0.097	1.12	1.08 - 1.17	9.11E-09	0.096
**Cognition**										
Cognition	Cognition	ACEs	1.25	1.19 - 1.31	2.78E-21	0.085	1.29	1.23 - 1.36	2.65E-23	0.060
Cognition	Cognition	PRS-SCZ	1.15	1.1 - 1.21	3.13E-09	0.085	1.17	1.11 - 1.23	4.22E-09	0.060
Cognition	Fluid intelligence impairment	ACEs	1.29	1.22 - 1.35	4.15E-24	0.087	1.33	1.26 - 1.41	1.14E-25	0.050
Cognition	Fluid intelligence impairment	PRS-SCZ	1.18	1.12 - 1.24	2.22E-10	0.087	1.19	1.12 - 1.26	1.26E-09	0.050
Cognition	Prospective memory impairment	ACEs	1.25	1.18 - 1.33	9.32E-13	0.063	1.31	1.22 - 1.41	5.12E-14	0.026
Cognition	Prospective memory impairment	PRS-SCZ	1.13	1.06 - 1.21	2.05E-04	0.063	1.13	1.05 - 1.22	1.14E-03	0.026
**Mania**										
Mania	More talkative than usual during mania	ACEs					1.31	1.2 - 1.42	1.16E-10	0.014
Mania	More talkative than usual during mania	PRS-SCZ					1.17	1.07 - 1.29	8.35E-04	0.014

Table S5. Effects of PRS-BIP and ACEs on psychopathological symptoms in the joint model analysis.

OR_all = odds ratio among all individuals; CI_all = 95% confidence intervals among all individuals; R2_all = Nagelkerke R2 among all individuals; P_all = P value among all individuals; OR_onlyBritish = odds ratio after excluding non-British individuals; CI_onlyBritish = 95% confidence intervals after excluding non-British individuals; R2_onlyBritish = Nagelkerke R2 after excluding non-British individuals; P_onlyBritish = P value after excluding non-British individuals; BIP = bipolar disorder; PRS = polygenic risk score; ACEs = adverse childhood experiences. Effects were derived from joint models incorporating both PRS-BIP and ACEs, with all the covariates adjusted for.

Domain	Symptoms	Exposure	OR_all	CI_all	P_all	R2_all	OR_onlyBritish	CI_onlyBritish	P_onlyBritish	R2_onlyBritish
**Psychotic**										
Psychotic	Ever heard an un-real voice	ACEs	2.38	2.14 - 2.65	9.35E-57	0.044				
Psychotic	Ever heard an un-real voice	PRS-BIP	1.27	1.12 - 1.43	1.35E-04	0.044				
Psychotic	Ever seen an un-real vision	ACEs	2.18	2.01 - 2.37	2.46E-78	0.035	2.24	2.05 - 2.44	2.80E-71	0.036
Psychotic	Ever seen an un-real vision	PRS-BIP	1.19	1.08 - 1.3	3.15E-04	0.035	1.16	1.05 - 1.29	3.38E-03	0.036
Psychotic	Ever talked to a health professional about unusual or psychotic experiences	ACEs	1.7	1.44 - 2	1.45E-10	0.074	1.6	1.34 - 1.91	2.12E-07	0.067
Psychotic	Ever talked to a health professional about unusual or psychotic experiences	PRS-BIP	1.45	1.21 - 1.73	4.61E-05	0.074	1.43	1.17 - 1.73	4.15E-04	0.067
Psychotic	Psychotic	ACEs	2.22	2.07 - 2.37	6.56E-122	0.039	2.27	2.11 - 2.44	3.54E-110	0.040
Psychotic	Psychotic	PRS-BIP	1.19	1.11 - 1.29	3.83E-06	0.039	1.17	1.08 - 1.27	1.42E-04	0.040
**Mania**										
Mania	Ever had period extreme irritability	ACEs	2.05	1.98 - 2.13	0.00E+00	0.073	2.06	1.99 - 2.15	2.57E-289	0.072
Mania	Ever had period extreme irritability	PRS-BIP	1.12	1.08 - 1.17	2.47E-09	0.073	1.14	1.1 - 1.19	3.62E-10	0.072
Mania	Ever had period of mania / excitability	ACEs	2.45	2.28 - 2.63	1.46E-137	0.063	2.43	2.25 - 2.63	1.72E-111	0.058
Mania	Ever had period of mania / excitability	PRS-BIP	1.29	1.19 - 1.39	3.33E-10	0.063	1.28	1.17 - 1.4	2.81E-08	0.058
Mania	Mania	ACEs	2.05	1.98 - 2.12	0.00E+00	0.072	2.05	1.98 - 2.13	7.04E-294	0.071
Mania	Mania	PRS-BIP	1.13	1.09 - 1.18	8.93E-11	0.072	1.15	1.1 - 1.2	2.08E-11	0.071
Mania	More confident than usual during mania	ACEs	1.24	1.13 - 1.35	1.88E-06	0.019	1.21	1.1 - 1.33	1.28E-04	0.018
Mania	More confident than usual during mania	PRS-BIP	1.22	1.11 - 1.34	6.51E-05	0.019	1.22	1.1 - 1.36	2.45E-04	0.018
Mania	More creative or had more ideas than usual during mania	PRS-BIP	1.35	1.22 - 1.49	5.66E-09	0.027	1.37	1.23 - 1.54	2.50E-08	0.027
Mania	More creative or had more ideas than usual during mania	ACEs	1.33	1.21 - 1.46	1.25E-09	0.027	1.32	1.19 - 1.46	1.44E-07	0.027
Mania	More talkative than usual during mania	ACEs	1.31	1.22 - 1.41	5.09E-13	0.014	1.31	1.21 - 1.42	1.03E-10	0.014
Mania	More talkative than usual during mania	PRS-BIP	1.2	1.1 - 1.3	2.13E-05	0.014	1.2	1.09 - 1.31	1.23E-04	0.014
Mania	Needed less sleep than usual during mania	ACEs	1.36	1.25 - 1.48	3.35E-13	0.015	1.36	1.24 - 1.49	4.85E-11	0.014
Mania	Needed less sleep than usual during mania	PRS-BIP	1.2	1.09 - 1.32	1.62E-04	0.015	1.19	1.08 - 1.32	8.40E-04	0.014
Mania	Severity of problems due to mania or irritability	ACEs	1.73	1.62 - 1.85	1.21E-61	0.038	1.75	1.63 - 1.88	2.83E-54	0.037
Mania	Severity of problems due to mania or irritability	PRS-BIP	1.21	1.13 - 1.3	2.66E-07	0.038	1.22	1.13 - 1.32	5.56E-07	0.037
**Depression**										
Depression	Depression	ACEs	1.77	1.71 - 1.84	2.28E-200	0.068	1.79	1.72 - 1.87	4.30E-174	0.068
Depression	Depression	PRS-BIP	1.15	1.11 - 1.19	2.27E-14	0.068	1.17	1.12 - 1.21	5.97E-15	0.068
Depression	Ever had prolonged feelings of sadness or depression	ACEs	1.93	1.86 - 2	1.66E-290	0.092	1.95	1.88 - 2.03	2.61E-251	0.093
Depression	Ever had prolonged feelings of sadness or depression	PRS-BIP	1.18	1.14 - 1.22	1.20E-20	0.092	1.2	1.16 - 1.25	5.16E-22	0.093
Depression	Ever had prolonged loss of interest in normal activities	ACEs	2.11	2.04 - 2.19	0.00E+00	0.103	2.11	2.04 - 2.19	0.00E+00	0.103
Depression	Ever had prolonged loss of interest in normal activities	PRS-BIP	1.18	1.14 - 1.22	8.95E-21	0.103	1.21	1.16 - 1.26	1.52E-22	0.103
Depression	Feelings of worthlessness during worst period of depression	ACEs	2.04	1.96 - 2.13	1.61E-223	0.082	2.03	1.94 - 2.13	5.49E-186	0.083
Depression	Feelings of worthlessness during worst period of depression	PRS-BIP	1.15	1.09 - 1.2	7.22E-09	0.082	1.16	1.11 - 1.22	2.67E-09	0.083
Depression	Impact on normal roles during worst period of depression	ACEs	1.69	1.62 - 1.77	2.55E-129	0.035	1.69	1.62 - 1.77	2.21E-110	0.035
Depression	Impact on normal roles during worst period of depression	PRS-BIP	1.11	1.06 - 1.16	1.74E-05	0.035	1.12	1.06 - 1.17	2.08E-05	0.035
Depression	Professional informed about depression	ACEs	1.63	1.56 - 1.7	3.23E-100	0.054	1.6	1.52 - 1.68	6.41E-79	0.050
Depression	Professional informed about depression	PRS-BIP	1.12	1.07 - 1.18	9.79E-07	0.054	1.11	1.05 - 1.16	7.57E-05	0.050
**Anxiety**										
Anxiety	Anxiety	ACEs	1.45	1.39 - 1.51	2.21E-62	0.018	1.42	1.35 - 1.49	7.43E-47	0.016

Anxiety	Anxiety	PRS-BIP	1.08	1.03 - 1.13	5.65E-04	0.018	1.09	1.04 - 1.15	2.81E-04	0.016
Anxiety	Ever felt worried, tense, or anxious for most of a month or longer	ACEs	1.96	1.89 - 2.04	1.91E-288	0.051	1.96	1.88 - 2.04	9.00E-242	0.050
Anxiety	Ever felt worried, tense, or anxious for most of a month or longer	PRS-BIP	1.17	1.12 - 1.21	3.01E-15	0.051	1.18	1.13 - 1.23	4.73E-15	0.050
Anxiety	Ever worried more than most people would in similar situation	ACEs	2.04	1.96 - 2.12	9.76E-284	0.069	2.04	1.95 - 2.13	9.54E-238	0.068
Anxiety	Ever worried more than most people would in similar situation	PRS-BIP	1.18	1.13 - 1.23	7.72E-15	0.069	1.18	1.13 - 1.24	1.41E-13	0.068
Anxiety	Multiple worries during worst period of anxiety	ACEs	1.53	1.43 - 1.63	1.02E-35	0.043	1.57	1.46 - 1.69	2.77E-34	0.046
Anxiety	Multiple worries during worst period of anxiety	PRS-BIP	1.16	1.08 - 1.24	4.09E-05	0.043	1.17	1.08 - 1.26	7.85E-05	0.046
**Help-seeking**										
Help-seeking	Ever sought or received professional help for mental distress	ACEs	2.03	1.97 - 2.1	0.00E+00	0.099	2.04	1.96 - 2.11	0.00E+00	0.095
Help-seeking	Ever sought or received professional help for mental distress	PRS-BIP	1.19	1.15 - 1.24	2.65E-23	0.099	1.2	1.16 - 1.25	6.14E-22	0.095
**Self-harm or suicide**										
Self-harm or suicide	Ever contemplated self-harm	ACEs	2.75	2.64 - 2.86	0.00E+00	0.105	2.77	2.65 - 2.9	0.00E+00	0.107
Self-harm or suicide	Ever contemplated self-harm	PRS-BIP	1.18	1.13 - 1.24	2.14E-12	0.105	1.21	1.15 - 1.28	4.06E-14	0.107
Self-harm or suicide	Ever self-harmed	ACEs	3.17	2.96 - 3.39	7.08E-250	0.108	3.18	2.95 - 3.42	1.41E-210	0.108
Self-harm or suicide	Ever self-harmed	PRS-BIP	1.26	1.16 - 1.36	5.72E-09	0.108	1.31	1.2 - 1.42	5.72E-10	0.108
Self-harm or suicide	Ever thought that life not worth living	ACEs	2.5	2.41 - 2.58	0.00E+00	0.093	2.51	2.42 - 2.61	0.00E+00	0.092
Self-harm or suicide	Ever thought that life not worth living	PRS-BIP	1.12	1.08 - 1.16	1.22E-09	0.093	1.14	1.1 - 1.19	3.67E-11	0.092
Self-harm or suicide	Self harm	ACEs	2.52	2.43 - 2.6	0.00E+00	0.097	2.53	2.44 - 2.63	0.00E+00	0.096
Self-harm or suicide	Self harm	PRS-BIP	1.13	1.09 - 1.17	4.87E-11	0.097	1.15	1.11 - 1.2	2.07E-12	0.096
**Cognition**										
Cognition	Cognition	ACEs	1.25	1.19 - 1.31	1.14E-21	0.085	1.29	1.23 - 1.36	1.01E-23	0.059
Cognition	Cognition	PRS-BIP	1.1	1.05 - 1.15	8.72E-05	0.085	1.09	1.03 - 1.15	1.19E-03	0.059
Cognition	Prospective memory impairment	ACEs	1.25	1.18 - 1.33	7.41E-13	0.063	1.31	1.22 - 1.41	4.31E-14	0.026
Cognition	Prospective memory impairment	PRS-BIP	1.13	1.06 - 1.21	2.34E-04	0.063	1.13	1.05 - 1.22	7.62E-04	0.026
**Psychotic**										
Psychotic	Distress caused by unusual or psychotic experiences	ACEs					1.46	1.27 - 1.69	2.93E-07	0.050
Psychotic	Distress caused by unusual or psychotic experiences	PRS-BIP					1.34	1.14 - 1.58	5.18E-04	0.050
Psychotic	Ever believed in an un-real conspiracy against self	ACEs					3.38	2.83 - 4.03	1.10E-41	0.073
Psychotic	Ever believed in an un-real conspiracy against self	PRS-BIP					1.38	1.13 - 1.68	1.45E-03	0.073
Mania										
Mania	More active than usual during mania	ACEs					1.34	1.24 - 1.45	1.41E-12	0.013
Mania	More active than usual during mania	PRS-BIP					1.17	1.07 - 1.28	7.90E-04	0.013
**Depression**										
Depression	Thoughts of death during worst depression	ACEs					1.49	1.42 - 1.56	2.03E-63	0.033
Depression	Thoughts of death during worst depression	PRS-BIP					1.08	1.03 - 1.13	2.57E-03	0.033

Table S6. Additive interaction effects of PRS-SCZ and ACEs on psychopathological symptoms.

RERI_all, relative excess risk due to interaction among all individuals; RERI_onlyBritish, relative excess risk due to interaction after excluding non-British individuals; PRS = polygenic risk score; ACEs = adverse childhood experiences. Effects were derived from logistic regression models incorporating both PRS-SCZ and ACEs, with all the covariates adjusted for. High PRS group was conposed of individuals in the top 25 percentile of PRS-SCZ.

Domain	Symptoms	RERI_all	CI_RERI_all	RERI_P_all	RERI_onlyBritish	CI_RERI_onlyBritish	RERI_P_onlyBritish
**Psychotic**							
Psychotic	Distress caused by unusual or psychotic experiences	-0.03	-0.54 - 0.47	0.895	-0.17	-0.74 - 0.41	0.569
Psychotic	Ever believed in an un-real conspiracy against self	-0.52	-1.61 - 0.57	0.347	-0.37	-1.68 - 0.94	0.583
Psychotic	Ever talked to a health professional about unusual or psychotic experiences	-0.09	-0.75 - 0.56	0.781	-0.6	-1.34 - 0.14	0.110
Psychotic	Psychotic	-0.04	-0.35 - 0.26	0.779			
**Mania**							
Mania	Ever had period extreme irritability	-0.01	-0.16 - 0.15	0.929	0.04	-0.14 - 0.23	0.652
Mania	Ever had period of mania / excitability	0	-0.36 - 0.35	0.986	0.1	-0.32 - 0.52	0.631
Mania	Mania	-0.01	-0.17 - 0.14	0.890	0.03	-0.15 - 0.21	0.767
Mania	More confident than usual during mania	-0.06	-0.33 - 0.2	0.649	-0.05	-0.35 - 0.25	0.745
Mania	More creative or had more ideas than usual during mania	-0.09	-0.38 - 0.2	0.556	-0.04	-0.38 - 0.3	0.820
Mania	More talkative than usual during mania				0.06	-0.2 - 0.32	0.646
**Depression**							
Depression	Depression	-0.03	-0.18 - 0.12	0.712	0.03	-0.15 - 0.22	0.728
Depression	Ever had prolonged feelings of sadness or depression	0.08	-0.09 - 0.24	0.351	0.16	-0.04 - 0.36	0.121
Depression	Ever had prolonged loss of interest in normal activities	0.09	-0.08 - 0.26	0.294	0.13	-0.07 - 0.32	0.215
Depression	Feelings of worthlessness during worst period of depression	0.07	-0.14 - 0.27	0.523	0.02	-0.21 - 0.25	0.858
Depression	Impact on normal roles during worst period of depression	0.06	-0.11 - 0.22	0.500	0.02	-0.16 - 0.2	0.827
Depression	Thoughts of death during worst depression	0.06	-0.09 - 0.21	0.413	0.05	-0.12 - 0.22	0.581
**Anxiety**							
Anxiety	Anxiety	0.14	-0.01 - 0.29	0.075	0.17	-0.01 - 0.34	0.063
Anxiety	Ever felt worried, tense, or anxious for most of a month or longer	0.2	0.03 - 0.37	0.021	0.21	0.02 - 0.41	0.033
Anxiety	Ever worried more than most people would in similar situation	0.13	-0.05 - 0.32	0.145	0.13	-0.08 - 0.34	0.230
**Help-seeking**							
Help-seeking	Ever sought or received professional help for mental distress	0.24	0.07 - 0.4	0.005	0.29	0.09 - 0.48	0.004
**Self-harm or suicide**							
Self-harm or suicide	Ever contemplated self-harm	0.02	-0.22 - 0.25	0.889	0.08	-0.19 - 0.36	0.555
Self-harm or suicide	Ever self-harmed	0.21	-0.2 - 0.62	0.315	0.06	-0.42 - 0.53	0.819
Self-harm or suicide	Ever thought that life not worth living	0	-0.18 - 0.19	0.975	0.07	-0.15 - 0.29	0.545
Self-harm or suicide	Self harm	0.02	-0.16 - 0.21	0.794	0.08	-0.14 - 0.3	0.488
**Cognition**							
Cognition	Cognition	-0.17	-0.3 - -0.03	0.015	-0.11	-0.28 - 0.05	0.177
Cognition	Fluid intelligence impairment	-0.23	-0.37 - -0.08	0.002	-0.16	-0.34 - 0.02	0.082
Cognition	Prospective memory impairment	-0.1	-0.28 - 0.07	0.233	-0.2	-0.42 - 0.03	0.089

Table S7. Additive interaction effects of PRS-BIP and ACEs on psychopathological symptoms.

RERI_all, relative excess risk due to interaction among all individuals; RERI_onlyBritish, relative excess risk due to interaction after excluding non-British individuals; PRS = polygenic risk score; ACEs = adverse childhood experiences. Effects were derived from logistic regression models incorporating both PRS-BIP and ACEs, with all the covariates adjusted for. High PRS group was conposed of individuals in the top 25 percentile of PRS-BIP.

Domain	Symptoms	RERI_all	CI_RERI_all	RERI_P_all	RERI_onlyBritish	CI_RERI_onlyBritish	RERI_P_onlyBritish
**Psychotic**							
Psychotic	Ever heard an un-real voice	-0.12	-0.66 - 0.42	6.63E-01			
Psychotic	Ever seen an un-real vision	-0.01	-0.39 - 0.36	9.39E-01	0.18	-0.26 - 0.61	4.27E-01
Psychotic	Ever talked to a health professional about unusual or psychotic experiences	0.42	-0.23 - 1.07	2.08E-01	0.32	-0.39 - 1.03	3.78E-01
Psychotic	Psychotic	-0.12	-0.44 - 0.19	4.32E-01	-0.02	-0.38 - 0.34	9.23E-01
Psychotic	Distress caused by unusual or psychotic experiences				0.19	-0.37 - 0.75	5.09E-01
Psychotic	Ever believed in an un-real conspiracy against self				0.28	-0.98 - 1.54	6.61E-01
**Mania**							
Mania	Ever had period extreme irritability	0.03	-0.13 - 0.19	6.96E-01	0.11	-0.09 - 0.3	2.76E-01
Mania	Ever had period of mania / excitability	-0.02	-0.39 - 0.35	9.23E-01	0.04	-0.39 - 0.46	8.71E-01
Mania	Mania	0.05	-0.11 - 0.21	5.52E-01	0.14	-0.06 - 0.33	1.63E-01
Mania	More confident than usual during mania	-0.12	-0.38 - 0.13	3.52E-01	-0.16	-0.46 - 0.13	2.79E-01
Mania	More creative or had more ideas than usual during mania	-0.09	-0.39 - 0.21	5.67E-01	-0.02	-0.38 - 0.33	8.95E-01
Mania	More talkative than usual during mania	-0.03	-0.25 - 0.2	8.01E-01	-0.01	-0.27 - 0.24	9.14E-01
Mania	Needed less sleep than usual during mania	0.09	-0.16 - 0.35	4.76E-01	0.19	-0.11 - 0.48	2.14E-01
Mania	Severity of problems due to mania or irritability	0.07	-0.18 - 0.33	5.64E-01	0.06	-0.23 - 0.35	6.84E-01
Mania	More active than usual during mania				0	-0.26 - 0.25	9.71E-01
**Depression**							
Depression	Depression	0.03	-0.12 - 0.19	6.71E-01	0.09	-0.1 - 0.29	3.40E-01
Depression	Ever had prolonged feelings of sadness or depression	0.15	-0.02 - 0.32	7.45E-02	0.27	0.06 - 0.47	1.24E-02
Depression	Ever had prolonged loss of interest in normal activities	0.25	0.08 - 0.42	4.70E-03	0.3	0.09 - 0.51	4.68E-03
Depression	Feelings of worthlessness during worst period of depression	-0.01	-0.21 - 0.19	9.25E-01	-0.13	-0.36 - 0.09	2.42E-01
Depression	Impact on normal roles during worst period of depression	0.09	-0.07 - 0.25	2.80E-01	0.13	-0.06 - 0.31	1.70E-01
Depression	Professional informed about depression	0.2	0.02 - 0.37	2.67E-02	0.15	-0.04 - 0.35	1.23E-01
Depression	Thoughts of death during worst depression				0.04	-0.12 - 0.2	6.38E-01
**Anxiety**							
Anxiety	Anxiety	0.11	-0.03 - 0.26	1.35E-01	0.18	0 - 0.35	4.66E-02
Anxiety	Ever felt worried, tense, or anxious for most of a month or longer	0.26	0.09 - 0.43	2.47E-03	0.33	0.13 - 0.53	1.13E-03
Anxiety	Ever worried more than most people would in similar situation	0.22	0.03 - 0.4	2.14E-02	0.22	0.01 - 0.44	4.38E-02
Anxiety	Multiple worries during worst period of anxiety	0	-0.24 - 0.24	9.97E-01	0.01	-0.28 - 0.29	9.61E-01
**Help-seeking**							
Help-seeking	Ever sought or received professional help for mental distress	0.23	0.06 - 0.4	6.88E-03	0.28	0.08 - 0.48	5.27E-03
**Self-harm or suicide**							
Self-harm or suicide	Ever contemplated self-harm	0.2	-0.04 - 0.44	1.07E-01	0.27	-0.01 - 0.55	6.25E-02
Self-harm or suicide	Ever self-harmed	0.35	-0.07 - 0.78	1.06E-01	0.37	-0.14 - 0.87	1.53E-01
Self-harm or suicide	Ever thought that life not worth living	0.13	-0.06 - 0.32	1.91E-01	0.18	-0.05 - 0.4	1.21E-01
Self-harm or suicide	Self harm	0.18	-0.01 - 0.37	6.83E-02	0.2	-0.03 - 0.43	8.11E-02
**Cognition**							
Cognition	Cognition	-0.01	-0.14 - 0.13	9.31E-01	-0.01	-0.17 - 0.15	8.79E-01
Cognition	Prospective memory impairment	-0.07	-0.24 - 0.1	4.12E-01	-0.12	-0.34 - 0.11	3.13E-01

Table S8. Multiplicative interaction effects of PRS-SCZ and ACEs on psychopathological symptoms.

SE = standard error; SCZ = schizophrenia; PRS = polygenic risk score; ACEs = adverse childhood experiences; multiplicative_interaction_all = multiplicative interaction effects among all individuals; SE_all = standard error among all individuals; P_all =P value fot the multiplicative interaction among all individuals; multiplicative_interaction_onlyBritish = multiplicative interaction effects after excluding non-British individuals; SE_onlyBritish = standard error after excluding non-British individuals; P_onlyBritish =P value after excluding non-British individuals. Effects were derived from models with both the main and interaction effects of PRS-SCZ and ACEs, with all the covariates adjusted for.

Domain	Symptoms	Exposure	multiplicative _interaction_ all	SE_all	P_all	multiplicative_interaction _only_British	SE_only_British	P_only_British
**PRS-SCZ × ACEs**								
**Psychotic**								
Psychotic	Psychotic	PRS-SCZ × ACEs	-0.543	0.175	1.92E-03	-0.501	0.201	1.28E-02
Psychotic	Ever believed in an un-real conspiracy against self	PRS-SCZ × ACEs	-0.101	0.078	1.92E-01			
Psychotic	Ever talked to a health professional about unusual or psychotic experiences	PRS-SCZ × ACEs	-0.213	0.181	2.38E-01	-0.480	0.206	1.97E-02
Psychotic	Distress caused by unusual or psychotic experiences	PRS-SCZ × ACEs	-0.133	0.155	3.90E-01	-0.210	0.176	2.34E-01
**Mania**								
Mania	Ever had period of mania / excitability	PRS-SCZ × ACEs	-0.146	0.080	6.85E-02	-0.100	0.093	2.82E-01
Mania	More creative or had more ideas than usual during mania	PRS-SCZ × ACEs	-0.123	0.103	2.31E-01	-0.082	0.121	4.97E-01
Mania	Mania	PRS-SCZ × ACEs	-0.047	0.042	2.61E-01	-0.022	0.048	6.52E-01
Mania	Ever had period extreme irritability	PRS-SCZ × ACEs	-0.042	0.042	3.26E-01	-0.012	0.049	8.12E-01
Mania	More confident than usual during mania	PRS-SCZ × ACEs	-0.091	0.098	3.52E-01	-0.075	0.115	5.12E-01
Mania	More talkative than usual during mania	PRS-SCZ × ACEs				0.006	0.098	9.50E-01
**Depression**								
Depression	Depression	PRS-SCZ × ACEs	-0.072	0.044	1.05E-01	-0.037	0.052	4.72E-01
Depression	Ever had prolonged loss of interest in normal activities	PRS-SCZ × ACEs	-0.044	0.041	2.78E-01	-0.031	0.047	5.02E-01
Depression	Ever had prolonged feelings of sadness or depression	PRS-SCZ × ACEs	-0.035	0.042	4.01E-01	0.002	0.050	9.73E-01
Depression	Feelings of worthlessness during worst period of depression	PRS-SCZ × ACEs	-0.026	0.052	6.16E-01	-0.050	0.060	3.98E-01
Depression	Impact on normal roles during worst period of depression	PRS-SCZ × ACEs	-0.021	0.050	6.79E-01	-0.039	0.057	4.90E-01
Depression	Thoughts of death during worst depression	PRS-SCZ × ACEs	0.007	0.050	8.83E-01	-0.003	0.058	9.63E-01
**Anxiety**								
Anxiety	Anxiety	PRS-SCZ × ACEs	0.054	0.051	2.96E-01	0.078	0.060	1.92E-01
Anxiety	Ever felt worried, tense, or anxious for most of a month or longer	PRS-SCZ × ACEs	0.015	0.043	7.29E-01	0.025	0.049	6.19E-01
Anxiety	Ever worried more than most people would in similar situation	PRS-SCZ × ACEs	-0.010	0.046	8.26E-01	-0.013	0.053	8.02E-01
**Help-seeking**								
Help-seeking	Ever sought or received professional help for mental distress	PRS-SCZ × ACEs	0.046	0.041	2.57E-01	0.067	0.047	1.54E-01
**Self-harm or suicide**								
Self-harm or suicide	Ever contemplated self-harm	PRS-SCZ × ACEs	-0.118	0.048	1.47E-02	-0.108	0.055	4.88E-02
Self-harm or suicide	Ever thought that life not worth living	PRS-SCZ × ACEs	-0.069	0.041	9.00E-02	-0.053	0.047	2.62E-01
Self-harm or suicide	Self harm	PRS-SCZ × ACEs	-0.060	0.041	1.40E-01	-0.048	0.047	3.01E-01
Self-harm or suicide	Ever self-harmed	PRS-SCZ × ACEs	-0.079	0.077	3.06E-01	-0.142	0.089	1.08E-01
**Cognition**								
Cognition	Fluid intelligence impairment	PRS-SCZ × ACEs	-0.204	0.055	2.29E-04	-0.159	0.066	1.70E-02
Cognition	Cognition	PRS-SCZ × ACEs	-0.157	0.053	3.18E-03	-0.119	0.063	5.69E-02
Cognition	Prospective memory impairment	PRS-SCZ × ACEs	-0.107	0.068	1.14E-01	-0.177	0.088	4.31E-02

Table S9. Multiplicative interaction effects of PRS-BIP and ACEs on psychopathological symptoms.
SE = standard error; BIP = bipolar disorder; PRS = polygenic risk score; ACEs = adverse childhood experiences; multiplicative_interaction_all = multiplicative interaction effects among all individuals; SE_all = standard error among all individuals; P_all =P value fot the multiplicative interaction among all individuals; multiplicative_interaction_onlyBritish = multiplicative interaction effects after excluding non-British individuals; SE_onlyBritish = standard error after excluding non-British individuals; P_onlyBritish =P value after excluding non-British individuals. Effects were derived from models with both the main and interaction effects of PRS-BIP and ACEs, with all the covariates adjusted for.

Domain	Symptoms	Exposure	multiplicative_interaction_all	SE_all	P_all	multiplicative_interaction_only_British	SE_only_British	P_only_British
**PRS-BIP × ACEs**								
**Psychotic**								
Psychotic	Psychotic	PRS-BIP × ACEs	-0.181	0.076	1.72E-02	-0.118	0.087	1.73E-01
Psychotic	Ever heard an un-real voice	PRS-BIP × ACEs	-0.229	0.120	5.71E-02			
Psychotic	Ever seen an un-real vision	PRS-BIP × ACEs	-0.120	0.094	1.99E-01	-0.013	0.106	8.99E-01
Psychotic	Ever talked to a health professional about unusual or psychotic experiences	PRS-BIP × ACEs	0.059	0.178	7.39E-01	0.038	0.201	8.51E-01
Psychotic	Distress caused by unusual or psychotic experiences	PRS-BIP × ACEs				0.019	0.173	9.13E-01
Psychotic	Ever believed in an un-real conspiracy against self	PRS-BIP × ACEs				-0.227	0.202	2.61E-01
**Mania**								
Mania	Ever had period of mania / excitability	PRS-BIP × ACEs	-0.195	0.079	1.30E-02	-0.163	0.092	7.46E-02
Mania	More creative or had more ideas than usual during mania	PRS-BIP × ACEs	-0.136	0.101	1.76E-01	-0.093	0.117	4.24E-01
Mania	More confident than usual during mania	PRS-BIP × ACEs	-0.131	0.097	1.80E-01	-0.158	0.113	1.62E-01
Mania	Ever had period extreme irritability	PRS-BIP × ACEs	-0.051	0.042	2.20E-01	-0.023	0.048	6.33E-01
Mania	Mania	PRS-BIP × ACEs	-0.047	0.041	2.54E-01	-0.012	0.048	8.05E-01
Mania	More talkative than usual during mania	PRS-BIP × ACEs	-0.067	0.083	4.23E-01	-0.054	0.096	5.70E-01
Mania	Severity of problems due to mania or irritability	PRS-BIP × ACEs	-0.048	0.074	5.21E-01	-0.062	0.084	4.58E-01
Mania	Needed less sleep than usual during mania	PRS-BIP × ACEs	0.015	0.093	8.68E-01	0.086	0.107	4.23E-01
Mania	More active than usual during mania	PRS-BIP × ACEs				-0.046	0.096	6.34E-01
**Depression**								
Depression	Feelings of worthlessness during worst period of depression	PRS-BIP × ACEs	-0.083	0.051	1.06E-01	-0.156	0.059	8.05E-03
Depression	Professional informed about depression	PRS-BIP × ACEs	0.074	0.053	1.61E-01	0.057	0.061	3.53E-01
Depression	Depression	PRS-BIP × ACEs	-0.045	0.044	3.09E-01	-0.021	0.052	6.87E-01
Depression	Ever had prolonged loss of interest in normal activities	PRS-BIP × ACEs	0.023	0.040	5.67E-01	0.031	0.047	5.04E-01
Depression	Ever had prolonged feelings of sadness or depression	PRS-BIP × ACEs	-0.007	0.042	8.66E-01	0.034	0.049	4.88E-01
Depression	Impact on normal roles during worst period of depression	PRS-BIP × ACEs	0.008	0.050	8.72E-01	0.029	0.056	6.01E-01
Depression	Thoughts of death during worst depression	PRS-BIP × ACEs				0.001	0.057	9.90E-01
**Anxiety**								
Anxiety	Ever felt worried, tense, or anxious for most of a month or longer	PRS-BIP × ACEs	0.051	0.043	2.29E-01	0.078	0.049	1.08E-01
Anxiety	Anxiety	PRS-BIP × ACEs	0.053	0.051	2.99E-01	0.095	0.059	1.10E-01
Anxiety	Multiple worries during worst period of anxiety	PRS-BIP × ACEs	-0.055	0.078	4.86E-01	-0.055	0.090	5.38E-01
Anxiety	Ever worried more than most people would in similar situation	PRS-BIP × ACEs	0.016	0.045	7.24E-01	0.015	0.052	7.78E-01
**Help-seeking**								
Help-seeking	Ever sought or received professional help for mental distress	PRS-BIP × ACEs	0.016	0.040	6.94E-01	0.034	0.046	4.61E-01
**Self-harm or suicide**								
Self-harm or suicide	Ever self-harmed	PRS-BIP × ACEs	-0.083	0.076	2.74E-01	-0.114	0.086	1.85E-01
Self-harm or suicide	Ever contemplated self-harm	PRS-BIP × ACEs	-0.049	0.048	3.07E-01	-0.044	0.054	4.19E-01
Self-harm or suicide	Ever thought that life not worth living	PRS-BIP × ACEs	-0.023	0.040	5.65E-01	-0.017	0.047	7.20E-01
Self-harm or suicide	Self harm	PRS-BIP × ACEs	-0.007	0.040	8.65E-01	-0.012	0.046	7.93E-01
**Cognition**								

Cognition	Prospective memory impairment	PRS-BIP × ACEs	-0.081	0.068	2.30E-01	-0.118	0.087	1.73E-01
Cognition	Cognition	PRS-BIP × ACEs	-0.024	0.053	6.53E-01	-0.029	0.062	6.37E-01

Table S10. Multiplicative interaction effects of PRSs and ACEs on psychopathological symptoms after adjusting for the interaction terms between covariates and PRSs or ACEs.

SE = standard error; BIP = bipolar disorder; SCZ = schizophrenia; PRS = polygenic risk score; ACEs = adverse childhood experiences.

Domain	Symptoms	Exposure	Estimate	SE	P
Psychotic	Psychotic	PRS-BIP × ACEs	-0.18	0.08	2.08E-02
Psychotic	Ever heard an un-real voice	PRS-BIP × ACEs	-0.18	0.12	1.44E-01
Psychotic	Ever seen an un-real vision	PRS-BIP × ACEs	-0.12	0.10	2.29E-01
Psychotic	Ever talked to a health professional about unusual or psychotic experiences	PRS-BIP × ACEs	0.10	0.18	5.96E-01
Mania	Ever had period of mania / excitability	PRS-BIP × ACEs	-0.18	0.08	2.68E-02
Mania	More creative or had more ideas than usual during mania	PRS-BIP × ACEs	-0.18	0.10	8.16E-02
Mania	More confident than usual during mania	PRS-BIP × ACEs	-0.16	0.10	1.14E-01
Mania	More talkative than usual during mania	PRS-BIP × ACEs	-0.08	0.09	3.45E-01
Mania	Ever had period extreme irritability	PRS-BIP × ACEs	-0.03	0.04	4.65E-01
Mania	Mania	PRS-BIP × ACEs	-0.03	0.04	5.35E-01
Mania	Severity of problems due to mania or irritability	PRS-BIP × ACEs	-0.03	0.08	7.13E-01
Mania	Needed less sleep than usual during mania	PRS-BIP × ACEs	-0.01	0.10	8.92E-01
Depression	Feelings of worthlessness during worst period of depression	PRS-BIP × ACEs	-0.08	0.05	1.08E-01
Depression	Professional informed about depression	PRS-BIP × ACEs	0.08	0.05	1.50E-01
Depression	Depression	PRS-BIP × ACEs	-0.04	0.04	3.20E-01
Depression	Ever had prolonged loss of interest in normal activities	PRS-BIP × ACEs	0.02	0.04	5.48E-01
Depression	Impact on normal roles during worst period of depression	PRS-BIP × ACEs	0.02	0.05	6.63E-01
Depression	Ever had prolonged feelings of sadness or depression	PRS-BIP × ACEs	-0.01	0.04	8.56E-01
Anxiety	Ever felt worried, tense, or anxious for most of a month or longer	PRS-BIP × ACEs	0.05	0.04	2.60E-01
Anxiety	Anxiety	PRS-BIP × ACEs	0.05	0.05	3.19E-01
Anxiety	Multiple worries during worst period of anxiety	PRS-BIP × ACEs	-0.05	0.08	5.58E-01
Anxiety	Ever worried more than most people would in similar situation	PRS-BIP × ACEs	0.02	0.05	6.80E-01
Help-seeking	Ever sought or received professional help for mental distress	PRS-BIP × ACEs	0.01	0.04	7.32E-01
Self-harm or suicide	Ever self-harmed	PRS-BIP × ACEs	-0.07	0.08	3.30E-01
Self-harm or suicide	Ever contemplated self-harm	PRS-BIP × ACEs	-0.04	0.05	3.50E-01
Self-harm or suicide	Ever thought that life not worth living	PRS-BIP × ACEs	-0.02	0.04	5.99E-01
Self-harm or suicide	Self harm	PRS-BIP × ACEs	-0.01	0.04	8.81E-01

Cognition	Prospective memory	PRS-BIP × ACEs	-0.08	0.07	2.56E-01
Cognition	Cognition	PRS-BIP × ACEs	-0.02	0.05	7.77E-01
Psychotic	Ever believed in an un-real conspiracy against self	PRS-SCZ × ACEs	-0.58	0.18	1.62E-03
Psychotic	Ever talked to a health professional about unusual or psychotic experiences	PRS-SCZ × ACEs	-0.23	0.19	2.23E-01
Psychotic	Psychotic	PRS-SCZ × ACEs	-0.09	0.08	2.40E-01
Psychotic	Distress caused by unusual or psychotic experiences	PRS-SCZ × ACEs	-0.17	0.16	2.92E-01
Mania	Ever had period of mania / excitability	PRS-SCZ × ACEs	-0.15	0.08	7.72E-02
Mania	More creative or had more ideas than usual during mania	PRS-SCZ × ACEs	-0.15	0.11	1.54E-01
Mania	More confident than usual during mania	PRS-SCZ × ACEs	-0.12	0.10	2.23E-01
Mania	Mania	PRS-SCZ × ACEs	-0.03	0.04	4.12E-01
Mania	Ever had period extreme irritability	PRS-SCZ × ACEs	-0.03	0.04	4.63E-01
Depression	Depression	PRS-SCZ × ACEs	-0.08	0.04	6.28E-02
Depression	Ever had prolonged loss of interest in normal activities	PRS-SCZ × ACEs	-0.05	0.04	2.26E-01
Depression	Ever had prolonged feelings of sadness or depression	PRS-SCZ × ACEs	-0.05	0.04	2.37E-01
Depression	Feelings of worthlessness during worst period of depression	PRS-SCZ × ACEs	-0.03	0.05	6.05E-01
Depression	Impact on normal roles during worst period of depression	PRS-SCZ × ACEs	-0.02	0.05	6.31E-01
Depression	Thoughts of death during worst depression	PRS-SCZ × ACEs	0.00	0.05	9.65E-01
Anxiety	Anxiety	PRS-SCZ × ACEs	0.05	0.05	3.79E-01
Anxiety	Ever worried more than most people would in similar situation	PRS-SCZ × ACEs	-0.02	0.05	6.90E-01
Anxiety	Ever felt worried, tense, or anxious for most of a month or longer	PRS-SCZ × ACEs	0.00	0.04	9.63E-01
Help-seeking	Ever sought or received professional help for mental distress	PRS-SCZ × ACEs	0.04	0.04	3.58E-01
Self-harm or suicide	Ever contemplated self-harm	PRS-SCZ × ACEs	-0.12	0.05	1.06E-02
Self-harm or suicide	Ever thought that life not worth living	PRS-SCZ × ACEs	-0.08	0.04	5.90E-02
Self-harm or suicide	Self harm	PRS-SCZ × ACEs	-0.07	0.04	9.56E-02
Self-harm or suicide	Ever self-harmed	PRS-SCZ × ACEs	-0.07	0.08	3.43E-01
Cognition	Fluid intelligence	PRS-SCZ × ACEs	-0.20	0.06	5.49E-04
Cognition	Cognition	PRS-SCZ × ACEs	-0.15	0.05	6.99E-03
Cognition	Prospective memory	PRS-SCZ × ACEs	-0.08	0.07	2.23E-01

Table S11. Effects of number of ACEs on psychopathological symptoms in the univariate models.
ACEs = adverse childhood experiences; OR = odds ratio; CI = 95% confidence intervals; R2 = Nagelkerke R2 .

Domain	Symptoms	Exposure	OR	CI	P	R2
Psychotic	Psychotic	5 ACEs vs. no ACE	6.14	4.15 - 9.08	1.22E-19	0.042
Psychotic	Psychotic	4 ACEs vs. no ACE	4.52	3.61 - 5.65	6.84E-40	0.042
Psychotic	Psychotic	3 ACEs vs. no ACE	2.93	2.51 - 3.43	6.59E-41	0.042
Psychotic	Psychotic	2 ACEs vs. no ACE	2.61	2.34 - 2.9	2.74E-68	0.042
Psychotic	Psychotic	1 ACE vs. no ACE	1.84	1.7 - 2	1.26E-48	0.042
Mania	Mania	5 ACEs vs. no ACE	4.3	3.04 - 6.07	1.39E-16	0.074
Mania	Mania	4 ACEs vs. no ACE	3.79	3.2 - 4.49	7.19E-54	0.074
Mania	Mania	3 ACEs vs. no ACE	2.93	2.65 - 3.24	2.16E-99	0.074
Mania	Mania	2 ACEs vs. no ACE	2.35	2.21 - 2.51	2.17E-153	0.074
Mania	Mania	1 ACE vs. no ACE	1.78	1.71 - 1.86	3.95E-158	0.074
Depression	Depression	4 ACEs vs. no ACE	2.91	2.3 - 3.69	8.26E-19	0.068
Depression	Depression	5 ACEs vs. no ACE	2.87	1.71 - 4.82	6.33E-05	0.068
Depression	Depression	3 ACEs vs. no ACE	2.51	2.21 - 2.84	2.17E-46	0.068
Depression	Depression	2 ACEs vs. no ACE	2.17	2.02 - 2.34	1.23E-93	0.068
Depression	Depression	1 ACE vs. no ACE	1.57	1.5 - 1.64	1.25E-92	0.068
Anxiety	Anxiety	3 ACEs vs. no ACE	1.72	1.5 - 1.97	1.83E-14	0.018
Anxiety	Anxiety	4 ACEs vs. no ACE	1.71	1.35 - 2.17	7.53E-06	0.018
Anxiety	Anxiety	2 ACEs vs. no ACE	1.6	1.48 - 1.74	6.53E-29	0.018
Anxiety	Anxiety	5 ACEs vs. no ACE	1.47	0.84 - 2.57	1.73E-01	0.018
Anxiety	Anxiety	1 ACE vs. no ACE	1.37	1.3 - 1.44	4.35E-33	0.018
Help-seeking	Ever sought or received professional help for mental distress	5 ACEs vs. no ACE	4.97	3.33 - 7.42	4.52E-15	0.101
Help-seeking	Ever sought or received professional help for mental distress	4 ACEs vs. no ACE	3.5	2.92 - 4.19	6.10E-42	0.101
Help-seeking	Ever sought or received professional help for mental distress	3 ACEs vs. no ACE	3.19	2.87 - 3.54	1.45E-104	0.101
Help-seeking	Ever sought or received professional help for mental distress	2 ACEs vs. no ACE	2.48	2.32 - 2.64	5.05E-169	0.101
Help-seeking	Ever sought or received professional help for mental distress	1 ACE vs. no ACE	1.74	1.67 - 1.81	3.83E-157	0.101
Self-harm or suicide	Self harm	5 ACEs vs. no ACE	9.21	5.99 - 14.15	4.24E-24	0.103
Self-harm or suicide	Self harm	4 ACEs vs. no ACE	5.83	4.85 - 7.02	8.75E-78	0.103
Self-harm or suicide	Self harm	3 ACEs vs. no ACE	4.49	4.04 - 4.98	3.25E-176	0.103
Self-harm or suicide	Self harm	2 ACEs vs. no ACE	3.1	2.91 - 3.31	1.92E-269	0.103
Self-harm or suicide	Self harm	1 ACE vs. no ACE	2.06	1.98 - 2.15	7.91E-267	0.103
Cognition	Cognition	5 ACEs vs. no ACE	2.16	1.38 - 3.39	8.20E-04	0.085
Cognition	Cognition	4 ACEs vs. no ACE	1.64	1.32 - 2.04	9.11E-06	0.085
Cognition	Cognition	3 ACEs vs. no ACE	1.47	1.29 - 1.68	1.35E-08	0.085
Cognition	Cognition	2 ACEs vs. no ACE	1.32	1.21 - 1.43	2.98E-10	0.085
Cognition	Cognition	1 ACE vs. no ACE	1.18	1.12 - 1.24	3.90E-09	0.085

Table S12. Joint effects of PRS-SCZ and number of ACEs on psychopathological symptoms.

ACEs = adverse childhood experiences; OR = odds ratio; CI = 95% confidence intervals; R2 = Nagelkerke R2 ; SCZ = schizophrenia;PRS = polygenic risk score.

Domain	Symptoms	Exposure	OR	CI	P	R2
Psychotic	Psychotic	5 ACEs vs. no ACE	6.11	4.12 - 9.04	1.60E-19	0.042
Psychotic	Psychotic	4 ACEs vs. no ACE	4.48	3.58 - 5.61	1.78E-39	0.042
Psychotic	Psychotic	3 ACEs vs. no ACE	2.92	2.49 - 3.42	1.82E-40	0.042
Psychotic	Psychotic	2 ACEs vs. no ACE	2.6	2.33 - 2.89	1.29E-67	0.042
Psychotic	Psychotic	1 ACE vs. no ACE	1.84	1.69 - 2	3.83E-48	0.042
Psychotic	Psychotic	PRS-SCZ	1.12	1.04 - 1.2	3.50E-03	0.042
Mania	Mania	5 ACEs vs. no ACE	4.29	3.04 - 6.06	1.47E-16	0.074
Mania	Mania	4 ACEs vs. no ACE	3.78	3.19 - 4.47	1.45E-53	0.074
Mania	Mania	3 ACEs vs. no ACE	2.92	2.65 - 3.23	6.26E-99	0.074
Mania	Mania	2 ACEs vs. no ACE	2.35	2.2 - 2.5	1.44E-152	0.074
Mania	Mania	1 ACE vs. no ACE	1.78	1.71 - 1.86	2.43E-157	0.074
Mania	Mania	PRS-SCZ	1.05	1.02 - 1.09	4.64E-03	0.074
Depression	Depression	4 ACEs vs. no ACE	2.9	2.29 - 3.67	1.24E-18	0.069
Depression	Depression	5 ACEs vs. no ACE	2.88	1.72 - 4.83	6.11E-05	0.069
Depression	Depression	3 ACEs vs. no ACE	2.5	2.2 - 2.83	5.63E-46	0.069
Depression	Depression	2 ACEs vs. no ACE	2.16	2.01 - 2.33	8.92E-93	0.069
Depression	Depression	1 ACE vs. no ACE	1.57	1.5 - 1.64	1.47E-91	0.069
Depression	Depression	PRS-SCZ	1.11	1.08 - 1.15	7.12E-10	0.069
Anxiety	Anxiety	3 ACEs vs. no ACE	1.7	1.48 - 1.96	4.80E-14	0.019
Anxiety	Anxiety	4 ACEs vs. no ACE	1.69	1.34 - 2.14	1.14E-05	0.019
Anxiety	Anxiety	2 ACEs vs. no ACE	1.59	1.47 - 1.73	2.45E-28	0.019
Anxiety	Anxiety	5 ACEs vs. no ACE	1.47	0.84 - 2.57	1.74E-01	0.019
Anxiety	Anxiety	1 ACE vs. no ACE	1.36	1.3 - 1.44	2.20E-32	0.019
Anxiety	Anxiety	PRS-SCZ	1.16	1.11 - 1.21	1.00E-11	0.019
Help-seeking	Ever sought or received professional help for mental distress	5 ACEs vs. no ACE	4.95	3.32 - 7.4	5.31E-15	0.102
Help-seeking	Ever sought or received professional help for mental distress	4 ACEs vs. no ACE	3.47	2.9 - 4.16	2.03E-41	0.102
Help-seeking	Ever sought or received professional help for mental distress	3 ACEs vs. no ACE	3.17	2.86 - 3.52	1.34E-103	0.102
Help-seeking	Ever sought or received professional help for mental distress	2 ACEs vs. no ACE	2.47	2.31 - 2.63	2.69E-167	0.102
Help-seeking	Ever sought or received professional help for mental distress	1 ACE vs. no ACE	1.73	1.66 - 1.8	1.80E-155	0.102
Help-seeking	Ever sought or received professional help for mental distress	PRS-SCZ	1.13	1.09 - 1.17	4.15E-12	0.102
Self-harm or suicide	Self harm	5 ACEs vs. no ACE	9.18	5.97 - 14.12	4.97E-24	0.103
Self-harm or suicide	Self harm	4 ACEs vs. no ACE	5.79	4.81 - 6.97	3.50E-77	0.103
Self-harm or suicide	Self harm	3 ACEs vs. no ACE	4.47	4.02 - 4.96	4.72E-175	0.103
Self-harm or suicide	Self harm	2 ACEs vs. no ACE	3.09	2.9 - 3.29	1.93E-267	0.103
Self-harm or suicide	Self harm	1 ACE vs. no ACE	2.06	1.98 - 2.14	7.61E-265	0.103
Self-harm or suicide	Self harm	PRS-SCZ	1.11	1.07 - 1.15	3.15E-09	0.103
Cognition	Cognition	5 ACEs vs. no ACE	2.15	1.37 - 3.38	8.75E-04	0.086
Cognition	Cognition	4 ACEs vs. no ACE	1.63	1.31 - 2.02	1.18E-05	0.086
Cognition	Cognition	3 ACEs vs. no ACE	1.46	1.28 - 1.67	2.81E-08	0.086
Cognition	Cognition	2 ACEs vs. no ACE	1.31	1.2 - 1.43	6.50E-10	0.086
Cognition	Cognition	1 ACE vs. no ACE	1.18	1.11 - 1.24	6.43E-09	0.086
Cognition	Cognition	PRS-SCZ	1.15	1.1 - 1.21	4.73E-09	0.086

Table S13. Joint effects of PRS-BIP and number of ACEs on psychopathological symptoms.
ACEs = adverse childhood experiences; OR = odds ratio; CI = 95% confidence intervals; R2 = Nagelkerke R2 ; BIP = bipolar disorder; PRS = polygenic risk score.

Domain	Symptoms	Exposure	OR	CI	P	R2
Psychotic	Psychotic	5 ACEs vs. no ACE	6.14	4.15 - 9.08	1.23E-19	0.043
Psychotic	Psychotic	4 ACEs vs. no ACE	4.47	3.58 - 5.6	2.14E-39	0.043
Psychotic	Psychotic	3 ACEs vs. no ACE	2.93	2.5 - 3.42	1.10E-40	0.043
Psychotic	Psychotic	2 ACEs vs. no ACE	2.6	2.33 - 2.89	1.36E-67	0.043
Psychotic	Psychotic	1 ACE vs. no ACE	1.84	1.7 - 2	2.21E-48	0.043
Psychotic	Psychotic	PRS-BIP	1.15	1.07 - 1.24	1.38E-04	0.043
Mania	Mania	5 ACEs vs. no ACE	4.3	3.04 - 6.08	1.35E-16	0.075
Mania	Mania	4 ACEs vs. no ACE	3.76	3.18 - 4.45	3.31E-53	0.075
Mania	Mania	3 ACEs vs. no ACE	2.93	2.65 - 3.23	4.99E-99	0.075
Mania	Mania	2 ACEs vs. no ACE	2.34	2.2 - 2.5	5.76E-152	0.075
Mania	Mania	1 ACE vs. no ACE	1.78	1.71 - 1.86	1.61E-157	0.075
Mania	Mania	PRS-BIP	1.13	1.09 - 1.17	6.79E-11	0.075
Depression	Depression	4 ACEs vs. no ACE	2.88	2.28 - 3.65	1.65E-18	0.069
Depression	Depression	5 ACEs vs. no ACE	2.88	1.72 - 4.83	6.08E-05	0.069
Depression	Depression	3 ACEs vs. no ACE	2.51	2.21 - 2.84	2.98E-46	0.069
Depression	Depression	2 ACEs vs. no ACE	2.16	2.01 - 2.33	6.94E-93	0.069
Depression	Depression	1 ACE vs. no ACE	1.57	1.5 - 1.64	2.81E-92	0.069
Depression	Depression	PRS-BIP	1.15	1.11 - 1.19	3.33E-15	0.069
Help-seeking	Ever sought or received professional help for mental distress	5 ACEs vs. no ACE	4.98	3.34 - 7.44	4.32E-15	0.103
Help-seeking	Ever sought or received professional help for mental distress	4 ACEs vs. no ACE	3.46	2.89 - 4.15	3.18E-41	0.103
Help-seeking	Ever sought or received professional help for mental distress	3 ACEs vs. no ACE	3.18	2.87 - 3.54	4.36E-104	0.103
Help-seeking	Ever sought or received professional help for mental distress	2 ACEs vs. no ACE	2.46	2.31 - 2.63	4.11E-167	0.103
Help-seeking	Ever sought or received professional help for mental distress	1 ACE vs. no ACE	1.73	1.67 - 1.81	2.29E-156	0.103
Help-seeking	Ever sought or received professional help for mental distress	PRS-BIP	1.18	1.14 - 1.22	5.91E-22	0.103
Self-harm or suicide	Self harm	5 ACEs vs. no ACE	9.22	6 - 14.18	4.12E-24	0.103
Self-harm or suicide	Self harm	4 ACEs vs. no ACE	5.79	4.81 - 6.97	3.96E-77	0.103
Self-harm or suicide	Self harm	3 ACEs vs. no ACE	4.48	4.04 - 4.97	9.67E-176	0.103
Self-harm or suicide	Self harm	2 ACEs vs. no ACE	3.09	2.9 - 3.3	9.59E-268	0.103
Self-harm or suicide	Self harm	1 ACE vs. no ACE	2.06	1.98 - 2.15	4.07E-266	0.103
Self-harm or suicide	Self harm	PRS-BIP	1.12	1.08 - 1.16	3.47E-10	0.103
Cognition	Cognition	5 ACEs vs. no ACE	2.15	1.37 - 3.38	8.58E-04	0.085
Cognition	Cognition	4 ACEs vs. no ACE	1.62	1.31 - 2.02	1.34E-05	0.085
Cognition	Cognition	3 ACEs vs. no ACE	1.47	1.29 - 1.68	1.65E-08	0.085
Cognition	Cognition	2 ACEs vs. no ACE	1.31	1.21 - 1.43	3.56E-10	0.085
Cognition	Cognition	1 ACE vs. no ACE	1.18	1.11 - 1.24	5.08E-09	0.085
Cognition	Cognition	PRS-BIP	1.1	1.05 - 1.15	1.12E-04	0.085

Table S14. Effects of types of ACEs on psychopathological symptoms in the univariate models.

ACEs = adverse childhood experiences; OR = odds ratio; CI = 95% confidence intervals; R2 = Nagelkerke R2.

Domain	Symptoms	Exposure	OR	CI	P	R2	significance
Psychotic	Psychotic	Physical abuse	2.05	1.88 - 2.24	1.75E-57	0.028	TRUE
Mania	Mania	Physical abuse	2.09	1.99 - 2.2	2.1E-181	0.059	TRUE
Depression	Depression	Physical abuse	1.66	1.57 - 1.76	7.23E-70	0.057	TRUE
Anxiety	Anxiety	Physical abuse	1.32	1.24 - 1.41	5.49E-17	0.013	TRUE
Help-seeking	Ever sought or received professional help for mental distress	Physical abuse	1.86	1.77 - 1.96	6.5E-130	0.082	TRUE
Self-harm or suicide	Self harm	Physical abuse	2.23	2.12 - 2.35	1.7E-218	0.070	TRUE
Cognition	Cognition	Physical abuse	1.25	1.16 - 1.33	1.76E-10	0.083	TRUE
Psychotic	Psychotic	Emotional abuse	2.67	2.48 - 2.88	1.3E-141	0.040	TRUE
Mania	Mania	Emotional abuse	2.3	2.2 - 2.4	2.8E-285	0.067	TRUE
Depression	Depression	Emotional abuse	2.2	2.09 - 2.32	3.2E-185	0.066	TRUE
Anxiety	Anxiety	Emotional abuse	1.68	1.58 - 1.78	8.84E-68	0.018	TRUE
Help-seeking	Ever sought or received professional help for mental distress	Emotional abuse	2.43	2.33 - 2.55	0	0.095	TRUE
Self-harm or suicide	Self harm	Emotional abuse	3.16	3.02 - 3.31	0	0.093	TRUE
Cognition	Cognition	Emotional abuse	1.16	1.09 - 1.23	2.71E-06	0.082	TRUE
Psychotic	Psychotic	Sexual abuse	2.16	1.93 - 2.42	9.06E-42	0.025	TRUE
Mania	Mania	Sexual abuse	1.85	1.72 - 1.98	1.89E-69	0.051	TRUE
Depression	Depression	Sexual abuse	1.66	1.53 - 1.8	3.54E-36	0.054	TRUE
Anxiety	Anxiety	Sexual abuse	1.29	1.18 - 1.41	2.08E-08	0.013	TRUE
Help-seeking	Ever sought or received professional help for mental distress	Sexual abuse	2.19	2.05 - 2.35	7.5E-110	0.081	TRUE
Self-harm or suicide	Self harm	Sexual abuse	2.31	2.16 - 2.47	1.5E-130	0.063	TRUE
Cognition	Cognition	Sexual abuse	1.23	1.12 - 1.35	1.06E-05	0.082	TRUE
Psychotic	Psychotic	Emotional neglect	2.06	1.87 - 2.26	1.58E-49	0.027	TRUE
Mania	Mania	Emotional neglect	2	1.89 - 2.12	2.9E-131	0.056	TRUE
Depression	Depression	Emotional neglect	1.95	1.83 - 2.08	9.62E-94	0.059	TRUE
Anxiety	Anxiety	Emotional neglect	1.51	1.4 - 1.62	8.33E-29	0.014	TRUE
Help-seeking	Ever sought or received professional help for mental distress	Emotional neglect	2.2	2.08 - 2.32	3.2E-168	0.085	TRUE
Self-harm or suicide	Self harm	Emotional neglect	2.97	2.81 - 3.14	0	0.077	TRUE
Cognition	Cognition	Emotional neglect	1.23	1.14 - 1.32	4.46E-08	0.082	
Psychotic	Psychotic	Physical neglect	1.64	1.41 - 1.91	9.78E-11	0.021	TRUE
Mania	Mania	Physical neglect	1.59	1.46 - 1.73	7.34E-27	0.048	TRUE
Depression	Depression	Physical neglect	1.23	1.12 - 1.34	5.08E-06	0.052	TRUE
Anxiety	Anxiety	Physical neglect	1.11	0.99 - 1.24	0.062797	0.012	
Help-seeking	Ever sought or received professional help for mental distress	Physical neglect	1.37	1.26 - 1.49	4E-14	0.075	TRUE
Self-harm or suicide	Self harm	Physical neglect	1.63	1.5 - 1.77	1.59E-31	0.057	TRUE
Cognition	Cognition	Physical neglect	2.16	1.94 - 2.4	4.99E-45	0.087	TRUE

Table S15. Effects of PRS-SCZ and types of ACEs on psychopathological symptoms in the joint models.

ACEs = adverse childhood experiences; OR = odds ratio; CI = 95% confidence intervals; R2 = Nagelkerke R2; SCZ = schizophrenia; PRS = polygenic risk score.

Exposure	OR	CI	P	R2	Symptoms
Physical abuse	2.05	1.87 - 2.23	9.24E-57	0.028	Psychotic
PRS-SCZ	1.14	1.06 - 1.23	4.15E-04	0.028	Psychotic
Physical abuse	2.09	1.99 - 2.2	2.57E-180	0.059	Mania
PRS-SCZ	1.07	1.03 - 1.11	2.17E-04	0.059	Mania
Physical abuse	1.66	1.56 - 1.75	4.11E-69	0.058	Depression
PRS-SCZ	1.12	1.09 - 1.16	1.83E-11	0.058	Depression
Physical abuse	1.32	1.24 - 1.41	1.58E-16	0.014	Anxiety
PRS-SCZ	1.16	1.12 - 1.21	7.96E-13	0.014	Anxiety
Physical abuse	1.86	1.77 - 1.95	3.20E-128	0.083	Help seeking
PRS-SCZ	1.14	1.11 - 1.18	4.10E-15	0.083	Help seeking
Physical abuse	2.23	2.12 - 2.34	2.08E-216	0.070	Self harm and suicide
PRS-SCZ	1.13	1.09 - 1.17	1.30E-12	0.070	Self harm and suicide
Physical abuse	1.24	1.16 - 1.33	3.66E-10	0.084	Cognition
PRS-SCZ	1.16	1.1 - 1.21	1.24E-09	0.084	Cognition
Emotional abuse	2.66	2.47 - 2.87	3.97E-140	0.040	Psychotic
PRS-SCZ	1.12	1.04 - 1.21	1.86E-03	0.040	Psychotic
Emotional abuse	2.29	2.19 - 2.4	1.60E-283	0.067	Mania
PRS-SCZ	1.06	1.02 - 1.1	1.33E-03	0.067	Mania
Emotional abuse	2.19	2.08 - 2.31	1.63E-183	0.067	Depression
PRS-SCZ	1.12	1.08 - 1.16	3.33E-10	0.067	Depression
Emotional abuse	1.67	1.58 - 1.77	2.32E-66	0.019	Anxiety
PRS-SCZ	1.16	1.11 - 1.21	9.59E-12	0.019	Anxiety
Emotional abuse	2.42	2.32 - 2.54	0.00E+00	0.096	Help seeking
PRS-SCZ	1.13	1.09 - 1.17	4.59E-13	0.096	Help seeking
Emotional abuse	3.15	3.01 - 3.3	0.00E+00	0.093	Self harm and suicide
PRS-SCZ	1.12	1.08 - 1.16	3.01E-10	0.093	Self harm and suicide
Emotional abuse	1.15	1.08 - 1.22	6.22E-06	0.083	Cognition
PRS-SCZ	1.16	1.1 - 1.21	2.38E-09	0.083	Cognition
Sexual abuse	2.15	1.92 - 2.41	3.27E-41	0.026	Psychotic
PRS-SCZ	1.15	1.07 - 1.24	1.31E-04	0.026	Psychotic
Sexual abuse	1.84	1.72 - 1.97	7.20E-69	0.051	Mania
PRS-SCZ	1.08	1.04 - 1.12	4.76E-05	0.051	Mania
Sexual abuse	1.65	1.53 - 1.79	1.08E-35	0.055	Depression
PRS-SCZ	1.13	1.09 - 1.17	1.06E-11	0.055	Depression
Sexual abuse	1.29	1.17 - 1.41	4.09E-08	0.014	Anxiety
PRS-SCZ	1.16	1.12 - 1.21	8.54E-13	0.014	Anxiety
Sexual abuse	2.18	2.04 - 2.34	1.06E-108	0.082	Help seeking
PRS-SCZ	1.15	1.11 - 1.18	1.72E-15	0.082	Help seeking
Sexual abuse	2.3	2.15 - 2.46	2.76E-129	0.064	Self harm and suicide
PRS-SCZ	1.14	1.1 - 1.18	2.40E-13	0.064	Self harm and suicide
Sexual abuse	1.23	1.12 - 1.34	1.59E-05	0.083	Cognition
PRS-SCZ	1.16	1.11 - 1.22	1.16E-09	0.083	Cognition

Emotional neglect	2.05	1.86 - 2.25	7.45E-49	0.027	Psychotic
PRS-SCZ	1.14	1.06 - 1.23	3.78E-04	0.027	Psychotic
Emotional neglect	2	1.89 - 2.11	2.15E-130	0.056	Mania
PRS-SCZ	1.07	1.03 - 1.11	1.74E-04	0.056	Mania
Emotional neglect	1.94	1.82 - 2.07	5.79E-93	0.060	Depression
PRS-SCZ	1.12	1.09 - 1.16	1.88E-11	0.060	Depression
Emotional neglect	1.5	1.4 - 1.62	3.49E-28	0.015	Anxiety
PRS-SCZ	1.16	1.12 - 1.21	1.49E-12	0.015	Anxiety
Emotional neglect	2.19	2.07 - 2.31	1.64E-166	0.086	Help seeking
PRS-SCZ	1.14	1.11 - 1.18	4.62E-15	0.086	Help seeking
Emotional neglect	2.96	2.8 - 3.13	0.00E+00	0.078	Self harm and suicide
PRS-SCZ	1.13	1.09 - 1.17	2.49E-12	0.078	Self harm and suicide
Emotional neglect	1.22	1.14 - 1.32	9.67E-08	0.083	Cognition
PRS-SCZ	1.16	1.1 - 1.21	1.64E-09	0.083	Cognition
Physical neglect	1.64	1.41 - 1.9	1.29E-10	0.022	Psychotic
PRS-SCZ	1.15	1.07 - 1.24	1.33E-04	0.022	Psychotic
Physical neglect	1.59	1.46 - 1.73	1.02E-26	0.048	Mania
PRS-SCZ	1.08	1.04 - 1.12	3.46E-05	0.048	Mania
Physical neglect	1.23	1.12 - 1.34	6.15E-06	0.053	Depression
PRS-SCZ	1.13	1.09 - 1.17	3.27E-12	0.053	Depression
Physical neglect	1.11	0.99 - 1.23	7.16E-02	0.013	Anxiety
PRS-SCZ	1.17	1.12 - 1.22	5.65E-13	0.013	Anxiety
Physical neglect	1.37	1.26 - 1.48	6.91E-14	0.076	Help seeking
PRS-SCZ	1.15	1.11 - 1.19	1.40E-16	0.076	Help seeking
Physical neglect	1.62	1.5 - 1.76	3.33E-31	0.058	Self harm and suicide
PRS-SCZ	1.14	1.1 - 1.18	2.56E-14	0.058	Self harm and suicide
Physical neglect	2.15	1.94 - 2.4	6.03E-45	0.088	Cognition
PRS-SCZ	1.16	1.1 - 1.21	1.57E-09	0.088	Cognition

Table S16. Effects of PRS-BIP and types of ACEs on psychopathological symptoms in the joint models.

ACEs = adverse childhood experiences; OR = odds ratio; CI = 95% confidence intervals; R2 = Nagelkerke R2; BIP = bipolar disorder; PRS = polygenic risk score.

Exposure	OR	CI	P	R2	Symptoms
Physical abuse	2,05	1.88 - 2.24	4.13E-57	0,029	Psychotic
PRS-BIP	1,17	1.09 - 1.26	1.51E-05	0,029	Psychotic
Physical abuse	2,09	1.99 - 2.2	1.75E-180	0,060	Mania
PRS-BIP	1,14	1.1 - 1.18	9.71E-13	0,060	Mania
Physical abuse	1,66	1.57 - 1.75	1.40E-69	0,058	Depression
PRS-BIP	1,15	1.12 - 1.19	1.54E-16	0,058	Depression
Physical abuse	1,32	1.24 - 1.41	6.90E-17	0,014	Anxiety
PRS-BIP	1,08	1.03 - 1.12	5.42E-04	0,014	Anxiety
Physical abuse	1,86	1.77 - 1.96	8.01E-129	0,084	Help seeking
PRS-BIP	1,19	1.15 - 1.23	9.81E-25	0,084	Help seeking
Physical abuse	2,23	2.12 - 2.34	1.84E-217	0,070	Self harm and suicide
PRS-BIP	1,13	1.09 - 1.17	1.14E-12	0,070	Self harm and suicide
Physical abuse	1,24	1.16 - 1.33	2.27E-10	0,08324	Cognition
PRS-BIP	1,1	1.05 - 1.15	5.80E-05	0,08324	Cognition
Emotional abuse	2,66	2.47 - 2.87	1.54E-140	0,040	Psychotic
PRS-BIP	1,16	1.08 - 1.25	9.16E-05	0,040	Psychotic
Emotional abuse	2,29	2.19 - 2.4	2.73E-283	0,067	Mania
PRS-BIP	1,13	1.09 - 1.17	2.76E-11	0,067	Mania
Emotional abuse	2,19	2.08 - 2.31	4.68E-184	0,067	Depression
PRS-BIP	1,15	1.11 - 1.19	1.55E-15	0,067	Depression
Emotional abuse	1,68	1.58 - 1.78	2.14E-67	0,018	Anxiety
PRS-BIP	1,07	1.03 - 1.12	1.20E-03	0,018	Anxiety
Emotional abuse	2,43	2.32 - 2.54	0.00E+00	0,097	Help seeking
PRS-BIP	1,18	1.14 - 1.22	1.16E-22	0,097	Help seeking
Emotional abuse	3,15	3.02 - 3.3	0.00E+00	0,093	Self harm and suicide
PRS-BIP	1,12	1.08 - 1.16	6.38E-11	0,093	Self harm and suicide
Emotional abuse	1,15	1.09 - 1.22	3.94E-06	0,08251	Cognition
PRS-BIP	1,1	1.05 - 1.15	8.21E-05	0,08251	Cognition
Sexual abuse	2,15	1.93 - 2.41	2.32E-41	0,026	Psychotic
PRS-BIP	1,18	1.1 - 1.27	8.68E-06	0,026	Psychotic
Sexual abuse	1,84	1.72 - 1.97	9.74E-69	0,052	Mania
PRS-BIP	1,14	1.1 - 1.18	7.95E-13	0,052	Mania
Sexual abuse	1,66	1.53 - 1.79	8.73E-36	0,055	Depression
PRS-BIP	1,15	1.11 - 1.19	4.74E-16	0,055	Depression
Sexual abuse	1,29	1.18 - 1.41	2.46E-08	0,013	Anxiety
PRS-BIP	1,08	1.03 - 1.12	6.22E-04	0,013	Anxiety
Sexual abuse	2,18	2.04 - 2.34	8.34E-109	0,082	Help seeking
PRS-BIP	1,19	1.15 - 1.23	2.36E-24	0,082	Help seeking
Sexual abuse	2,3	2.15 - 2.46	1.15E-129	0,064	Self harm and suicide
PRS-BIP	1,13	1.09 - 1.17	2.42E-12	0,064	Self harm and suicide
Sexual abuse	1,23	1.12 - 1.35	1.37E-05	0,08244	Cognition
PRS-BIP	1,1	1.05 - 1.15	5.88E-05	0,08244	Cognition

Emotional neglect	2,05	1.86 - 2.25	4.33E-49	0,027	Psychotic
PRS-BIP	1,17	1.09 - 1.26	2.22E-05	0,027	Psychotic
Emotional neglect	2	1.89 - 2.11	2.04E-130	0,057	Mania
PRS-BIP	1,14	1.1 - 1.18	1.12E-12	0,057	Mania
Emotional neglect	1,95	1.83 - 2.07	3.16E-93	0,060	Depression
PRS-BIP	1,15	1.11 - 1.19	3.46E-16	0,060	Depression
Emotional neglect	1,51	1.4 - 1.62	1.18E-28	0,015	Anxiety
PRS-BIP	1,08	1.03 - 1.12	7.52E-04	0,015	Anxiety
Emotional neglect	2,19	2.07 - 2.32	5.47E-167	0,086	Help seeking
PRS-BIP	1,19	1.15 - 1.23	3.25E-24	0,086	Help seeking
Emotional neglect	2,96	2.8 - 3.13	0.00E+00	0,078	Self harm and suicide
PRS-BIP	1,13	1.09 - 1.17	4.25E-12	0,078	Self harm and suicide
Emotional neglect	1,23	1.14 - 1.32	6.52E-08	0,08278	Cognition
PRS-BIP	1,1	1.05 - 1.15	6.11E-05	0,08278	Cognition
Physical neglect	1,63	1.41 - 1.9	1.37E-10	0,022	Psychotic
PRS-BIP	1,18	1.09 - 1.27	1.15E-05	0,022	Psychotic
Physical neglect	1,58	1.46 - 1.72	1.47E-26	0,049	Mania
PRS-BIP	1,14	1.1 - 1.18	4.54E-13	0,049	Mania
Physical neglect	1,23	1.12 - 1.34	6.35E-06	0,053	Depression
PRS-BIP	1,15	1.12 - 1.19	1.59E-16	0,053	Depression
Physical neglect	1,11	0.99 - 1.24	6.75E-02	0,012	Anxiety
PRS-BIP	1,08	1.03 - 1.12	6.02E-04	0,012	Anxiety
Physical neglect	1,37	1.26 - 1.48	7.64E-14	0,076	Help seeking
PRS-BIP	1,19	1.15 - 1.23	2.40E-25	0,076	Help seeking
Physical neglect	1,62	1.5 - 1.76	3.31E-31	0,058	Self harm and suicide
PRS-BIP	1,14	1.1 - 1.18	3.38E-13	0,058	Self harm and suicide
Physical neglect	2,15	1.94 - 2.4	6.11E-45	0,08746	Cognition
PRS-BIP	1,1	1.05 - 1.15	9.31E-05	0,08746	Cognition

Table S17. Associations between PRSs of SCZ or BIPs and ACEs, with all covariates adjusted for.

PRS = polygenic risk score; OR = odds ratio; SCZ = schizophrenia; BIP = bipolar disorder; ACEs = adverse childhood experiences.

PRS	OR	Confidence interval	P value
PRS-SCZ	1.20	1.16 - 1.24	3.09E-28
PRS-BIP	1.11	1.08 - 1.15	9.66E-11
